# Chloroplasts evolved an additional layer of translational regulation based on non-AUG start codons for proteins with different turnover rates

**DOI:** 10.1038/s41598-022-27347-9

**Published:** 2023-01-17

**Authors:** Leelavathi Sadhu, Krishan Kumar, Saravanan Kumar, Abhishek Dass, Ranjana Pathak, Amit Bhardwaj, Pankaj Pandey, Nguyen Van Cuu, Bhupendra S. Rawat, Vanga Siva Reddy

**Affiliations:** 1 Plant Transformation Group, International Centre for Genetic Engineering and Biotechnology (ICGEB), New Delhi, 110067 India; 2 ICAR-Indian Institute of Maize Research, Delhi Unit, Pusa Campus, New Delhi, 110012 India; 3 Proteomics Facility, Thermo Fisher Scientific, Bangalore, Karnataka 560066 India; 4 Department of Microbiology & Immunology, Centre for Infectious Diseases, Stony Brook University, Stony Brook, New York, 11794 USA; 5 Perlmutter Cancer Center, NYU Langone Health, New York, 10016 USA; 6 Department of Plant Pathology, Institute of Agricultural Genetics (AGI), Hanoi, Vietnam

**Keywords:** Biotechnology, Molecular biology, Plant sciences

## Abstract

Chloroplasts have evolved from photosynthetic cyanobacteria-like progenitors through endosymbiosis. The chloroplasts of present-day land plants have their own transcription and translation systems that show several similarities with prokaryotic organisms. A remarkable feature of the chloroplast translation system is the use of non-AUG start codons in the protein synthesis of certain genes that are evolutionarily conserved from Algae to angiosperms. However, the biological significance of such use of non-AUG codons is not fully understood. The present study was undertaken to unravel the significance of non-AUG start codons in vivo using the chloroplast genetic engineering approach. For this purpose, stable transplastomic tobacco plants expressing a reporter gene i.e. *uidA* (GUS) under four different start codons (AUG/UUG/GUG/CUG) were generated and β-glucuronidase (GUS) expression was compared. To investigate further the role of promoter sequences proximal to the start codon, *uidA* was expressed under two different chloroplast gene promoters *psbA* and *psbC* that use AUG and a non-AUG (GUG) start codons, respectively, and also showed significant differences in the DNA sequence surrounding the start codon. Further, to delineate the role of RNA editing that creates AUG start codon by editing non-AUG codons, if any, which is another important feature of the chloroplast transcription and translation system, transcripts were sequenced. In addition, a proteomic approach was used to identify the translation initiation site(s) of GUS and the N-terminal amino acid encoded when expressed under different non-AUG start codons. The results showed that chloroplasts use non-AUG start codons in combination with the translation initiation site as an additional layer of gene regulation to over-express proteins that are required at high levels due to their high rates of turnover.

## Introduction

Chloroplasts are semi-autonomous organelles present in Algae to higher land plants and are generally considered to have originated from a photosynthetic cyanobacteria-like progenitor through endosymbiosis. Chloroplasts/plastids have their genome, although relatively small as compared to photosynthetic bacteria due to migration and subsequent integration of most of the genome into the nuclear genome^1^. The chloroplast genome of the present terrestrial plant codes for about 120–130 genes, mainly involved in photosynthesis, lipid metabolism, transcription, and translation mechanisms besides other cellular functions^2^. However, chloroplasts target the proteins encoded by nuclear-migrated genes for their functions through various mechanisms^3^. Due to the prokaryotic origin of chloroplasts, their transcription and translation closely resemble those of prokaryotic organisms, and studies have shown that chloroplast proteins can complement functionally in prokaryotes^4,5^. However, chloroplasts have evolved several other unique features involved in gene regulation at both transcription and translation stages^6–10^.

Similar to prokaryotes, translation initiation in chloroplasts is facilitated by the mRNA–rRNA interaction at the Shine–Dalgarno (SD) sequence and the key determinant in the process is the AUG start codon itself that interacts with N-formylmethionyl transfer RNA (tRNAfMet) to initiate the protein translation process^11,12^. Although AUG is the universal start codon for protein synthesis, several non-AUG codons have been shown to function as start codons^13–21^. For instance, ranslation of *infA* gene encoding initiation factor 1 has been shown to initiate from the UUG codon in green alga *Chlorella vulgaris* and tobacco^22^. Similarly, the translation of *psbC* gene in cyanobacterium *Synechocystis* has been shown to initiate from the GUG codon although an AUG codon was present just upstream of the GUG codon^23^. Such use of non-AUG codons is conserved across species and genera^24^, suggesting their biological significance. It has been estimated that more than ten *Arabidopsis* chloroplast genes are likely to be translated from non-AUG codons^25^. Recently, a systematic study conducted in *E. coli* involving the replacement of AUG with 63 non-AUG codons has shown that protein translation can initiate from a very large number of non-AUG codons, although the level of protein expression may vary significantly depending on the non-AUG codon used^26^. More recently, a proteomics-based approach has further revealed protein translation from non-canonical translation initiation sites (TIS) for several proteins in human cells^21^. In light of these findings, it is not clear whether transcription and translational mechanisms of the present-day terrestrial plant chloroplasts have evolved simply plasticity to accommodate any naturally occurring mutations or whether the chloroplasts used such evolved plasticity to select certain mutations to regulate gene expression further to meet the required levels of proteins for their functions.

The overall objective of this study is to unravel some of the regulatory features of transcription and translation mechanisms operating in the higher plant chloroplasts. For this purpose, systematic in vivo studies were conducted where the expression of the *uidA* (GUS) reporter gene was studied under the regulation of *psbC* promoter of tobacco that uses GUG as the start codon and compared its expression under four different start codons (AUG/UUG/GUG/CUG) that differed in their first base in the start codon. To ensure that the translation initiation context remained the same, the entire 5′ UTR and first six codons of *psbC* gene (codes for PSII 43KDa protein) were fused in frame with *uidA* gene. To assess further the role of sequence context surrounding the start codon in GUS expression, *uidA* with four different start codons (AUG/UUG/GUG/CUG) was also expressed under the regulation of a heterologous rice *psbA* (codes for PSII D1 protein) promoter that uses AUG as the start codon. In addition, to decipher the extent of changes that took place during the long course of evolution, all eight constructs tested for GUS expression in tobacco chloroplasts were also expressed in *E. coli* and compared the GUS protein expression/activity. The comparative GUS expression in tobacco chloroplasts suggests that plastid might have preferentially selected GUG over the universal AUG start codon to meet the required amount of PsbC protein, a component of the PSII complex that undergoes high turnover during photosynthesis. This view is further supported by the expression data of these constructs in *E. coli* where high levels of GUS were observed when AUG is the start codon, unlike the GUG start codon in chloroplasts. In addition, proteomics studies aimed at revealing protein translation initiation and N-terminal modifications showed that translation is initiated from multiple upstream sites in chloroplasts than from the predicted AUG/non-AUG sites. Another important observation was that the mRNA editing phenomenon commonly observed in chloroplasts that edit C base to U post transcription to create a start codon (ACG to AUG) did not alter any of the three non-AUG codons tested into an AUG codon. Taken together, all these pieces of experimental evidence underline significant evolutionary changes that chloroplasts of land plants have acquired to regulate the expression of selected genes to optimize the desired levels of protein using non-AUG start codons and other flanking sequences.

## Results

### psbC*:uidA* gene constructs designed to study protein expression under different start codons

**Figure 1:**
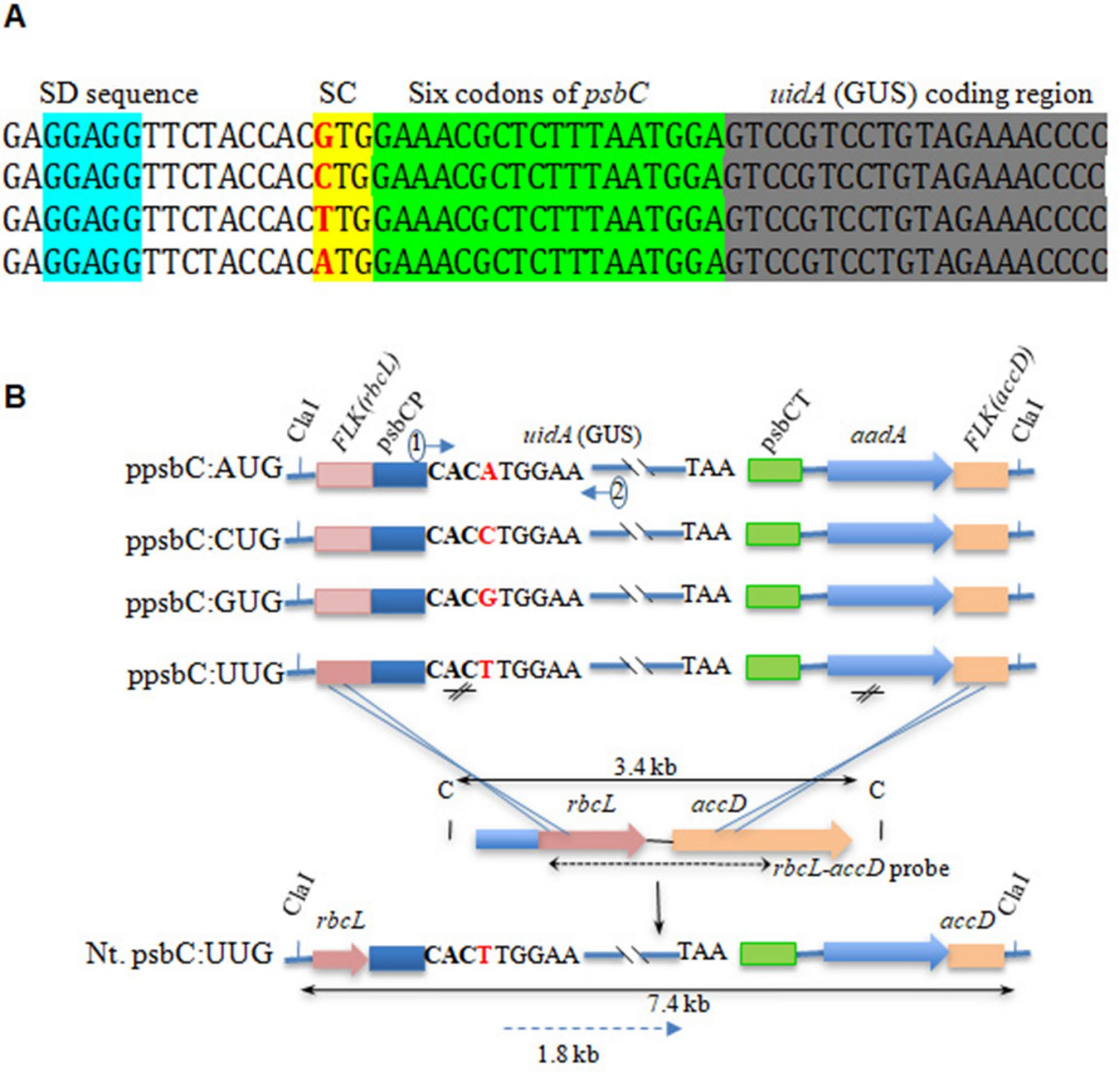
Schematic representation of expression cassettes used for chloroplast transformation. (**A**) Partial sequence of *uidA* (GUS) gene fused in frame with *psbC* gene 5′ untranslated region (5′UTR). SD and SC represent Shine–Dalgarno sequence (blue) and start codon (yellow), respectively. The first six codons (following start codon) are highlighted in green. (**B**) Partial restriction map showing the chimeric genes *uidA* and *aadA*. Also shown is the site of wild-type tobacco plastome where the chimeric *uidA* and *aadA* genes are expected to be integrated into the chloroplast genome (crossed lines) and the resulting transplastome with restriction sites and anticipated size of DNA fragments (solid line with double arrow) when restricted with ClaI restriction enzyme. Nt., psbCP, psbCT, FLK(*rbcL*), FLK(*aacD*) represent *Nicotiana tabacum,* psbC promoter, psbC terminator, partial sequences from *rbcL* and *accD* genes as flanking sequences (FLK) on either side of the transgene cassettes, respectively.

Four gene constructs that differed in the first nucleotide of the start codon (**A**UG/**G**UG/**C**UG/**U**UG) were created to study *uidA* expression under the tobacco *psbC* promoter (Fig. 1A). Based on previous studies by Kuroda and Maliga^27^ where it was shown that sequences downstream of the translation initiation codon play an important role in translation efficiency in chloroplasts, the sequence coding for the first six N-terminal amino acids of *psbC* was fused in frame with *uidA* to retain the translation initiation context of the native *psbC* (Fig. 1A). The tobacco partial *rbcL:accD* gene sequences were used to target site-specific integration of the chimeric *psbC:uidA* and selectable *aadA* genes through homologous recombination with the native plastid genome (Fig. 1B). The direction and the expected size of *psbC:uidA* transcripts and the location of the relevant restriction enzyme sites when stably integrated into the tobacco genome are shown in Fig. 1B.

### Site-specific integration of chimeric psbC:*uidA* gene into tobacco chloroplast genome

**Figure 2:**
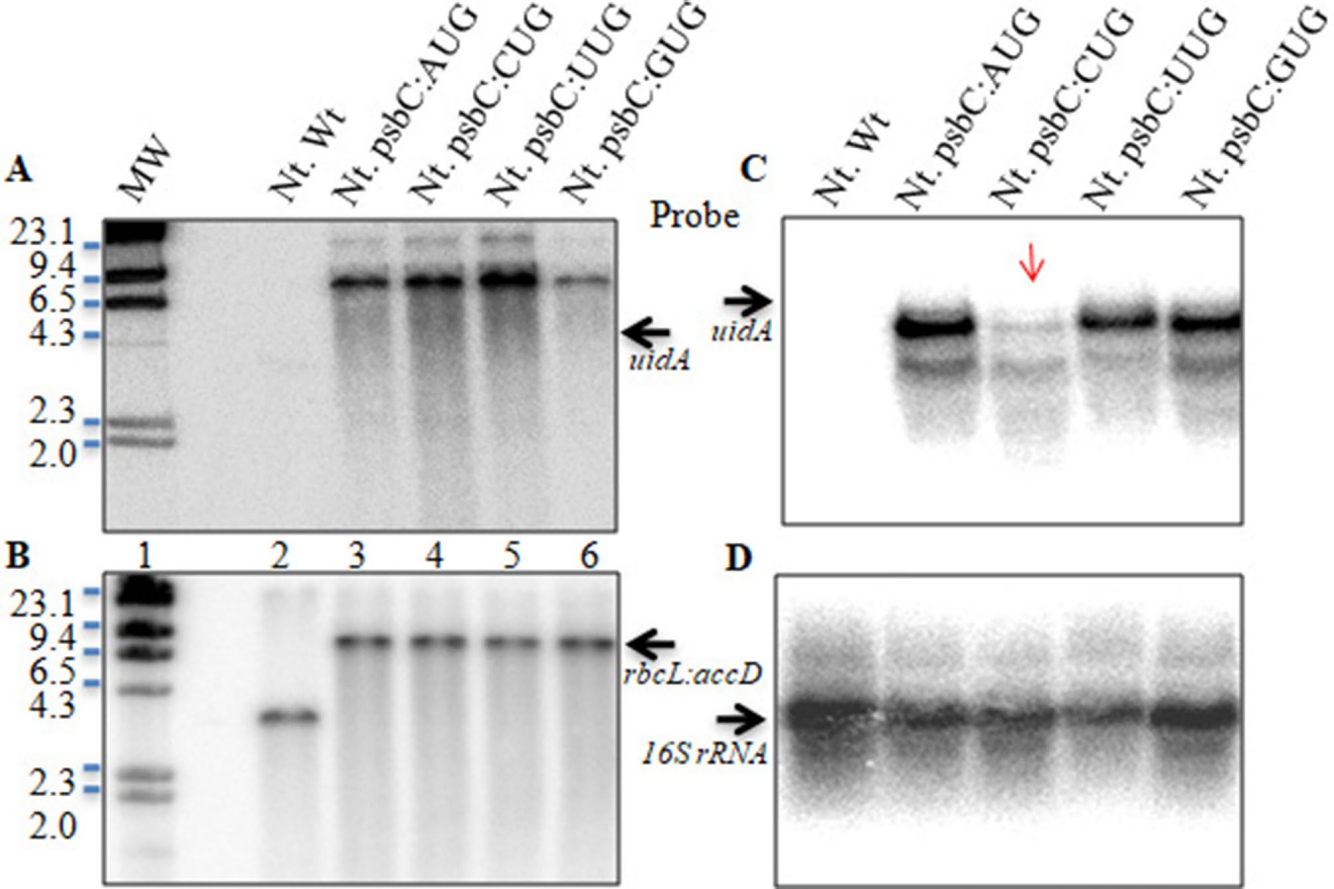
Stable integration and expression of GUS in tobacco chloroplasts under the tobacco psbC promoter with four different start codons. Southern hybridization of total genomic DNA probed with *uidA* (**A**) and partial gene sequences of *rbcL*:*accD* (**B**). Lane 1–6 correspond to size ladder (MW), wild type/control (Nt. Wt), psbC:AUG, psbC:CUG, psbC:TUG and psbC:GUG transplastomic *Nicotiana tabacum* (Nt.) plants, respectively. Note that in transcript/RNA Uracil (U) is used while in DNA/construct nucleotide Thymine (T) is used in place of Uracil. (**C**) Northern hybridization showing transcription of chimeric *uidA* under four different start codons in tobacco transplastomic plants. (**D**) The same blot reprobed with 16S rRNA to show equal loading of total RNA.

Southern hybridization showed the presence of ~ 7.4-kb band in the transplastomic plants (Fig. 2A) and not in wild-type plants when probed with the *uidA* gene sequence, which provided evidence for the stable and site-specific integration of psbC:*uidA* into the plastome (plastid/chloroplast genome). Furthermore, the presence of a 3.4 kb band in wild-type plants (Fig. 2B, lane 2) and its absence in transplastomic plants (Fig. 2B, lanes 3–6) when probed with *rbcL:accD* sequences further confirmed the stable integration of the transgene cassettes into the plastid genome. These results also showed the homoplasmic nature of transplastomes in the transformed plants that were selected for GUS expression and its activity studies.

### Transcription of psbC:*uidA* under *psbC* promoter with different start codons

Northern blot analysis showed the presence of *uidA* transcripts in all four transplastomic plants (Fig. 2C). In addition to the expected ~ 2.1 kb major band representing *uidA* transcript^28^, the presence of a minor band of lower size was also observed in all transplastomic lanes, which might represent the *uidA* transcripts that are not denatured completely at the time of gel loading. However, *uidA* transcript levels were significantly less where CUG was used as the start codon. To rule out any unequal loading of total RNA in the gel, the same blot was reprobed using the 16S rRNA sequence, and the results showed that RNA was loaded equally in all four lanes (Fig. 2D).

### Role of sequence context flanking start codon in transcription and translation

**Figure 3:**
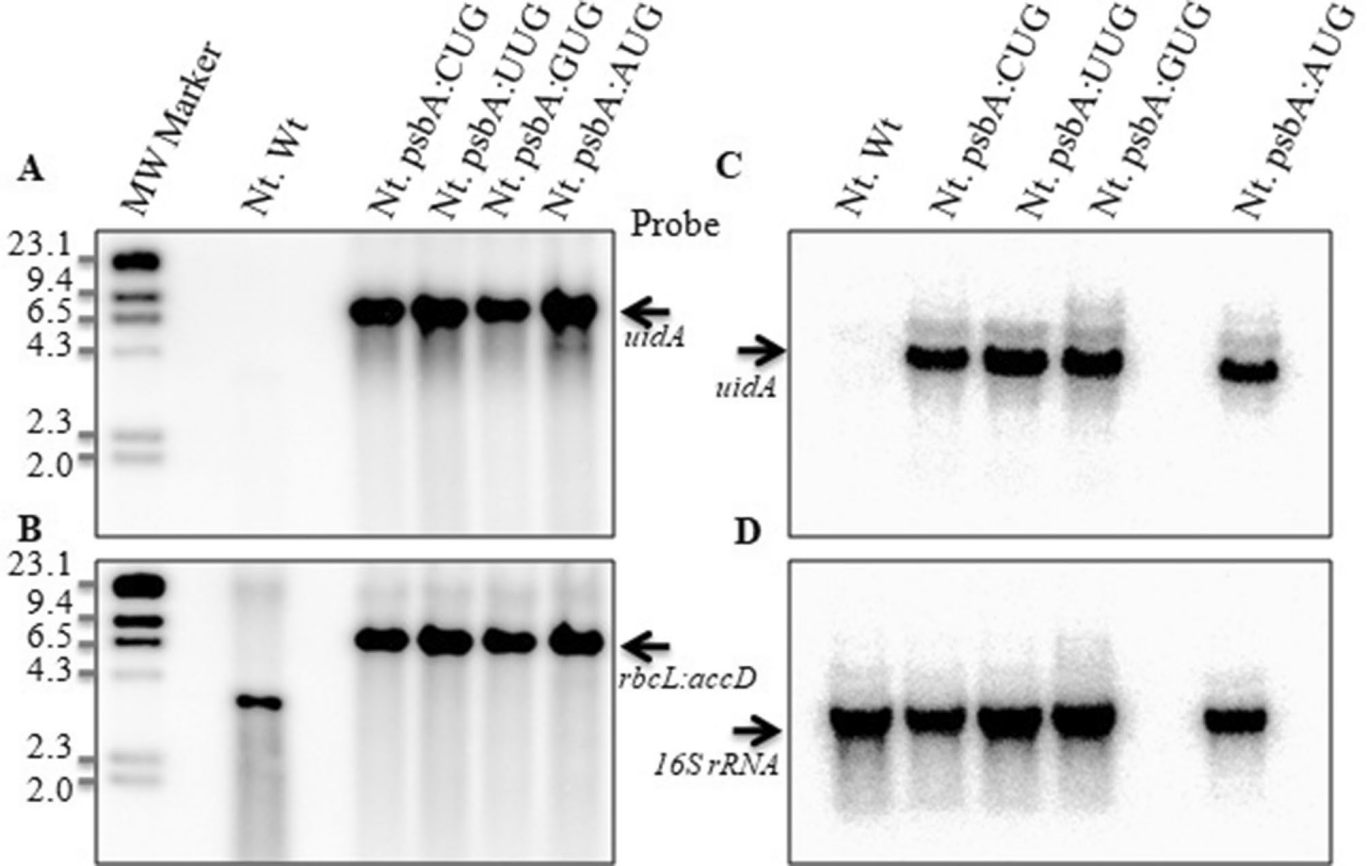
Stable site-specific integration and expression of *uidA* under the rice psbA promoter with different start codons in tobacco chloroplasts. Southern hybridization of genomic DNA using *uidA* (**A**) and partial *rbcL:accD* (**B**) gene sequences to show the site-specific integration of transgenes into tobacco plastome. Northern hybridization showing the transcription of chimeric *uidA* in four different transplastomic plants (**C**) and the blot probed with 16S rRNA to show equal loading of total RNA (**D**).

To assess the influence of the sequence around the start codon on transcription and protein synthesis, another set of four constructs was developed where *uidA* was placed under the heterologous rice chloroplast *psbA* promoter (Supplementary Fig. S1)^29^with different start codons (AUG, GUG, CUG and UUG) and stably transformed transplastomic tobacco plants were developed. The rice *psbA* promoter shared a high degree of homology with the tobacco *psbA* promoter (Supplementary Fig. S2A) and was shown to transcribe foreign genes efficiently in tobacco chloroplasts previously by our group^30^ and hence no fusions were made with *psbA* gene sequences coding N-terminal amino acids. The sequence homology between tobacco *psbC* and rice *psbA* promoters surrounding the start codon and 5′ UTR is shown in Supplementary Fig. S2B. While − 35 and − 10 sequences and ribosome binding site (SD) are well conserved between rice and tobacco for *psbA* promoters, the sequences between SD and the start codon differed significantly between *psbC* and *psbA* promoters (Supplementary Fig. S3). It is worth noting here that while AUG is the start codon for *psbA* in both rice^29^ and tobacco^31^, GUG is the start codon of *psbC* in tobacco^23^. Moreover, efficient expression of *uidA* gene under the heterologous rice *psbA* promoter in tobacco chloroplasts has been reported earlier^30^. Stable integration of transgene cassettes (*psbA:uidA* and *aadA*) was confirmed using Southern hybridization. An expected 7.4 kb band was observed in the transplastomic plants when probed with the *uidA* coding sequence (Fig. 3A). No corresponding band was observed in control plants, confirming the transplastomic nature of the plants regenerated under spectinomycin selection. Reprobing of the same blot with *rbcL:accD* flanking sequences further confirmed the site-specific integration of *uidA* and *aadA* genes into the tobacco plastome (Fig. 3B).

Northern blot analysis confirmed efficient transcription of *uidA* under the rice *psbA* promoter, and an expected 1.8 kb band corresponding to *uidA* transcripts was observed in all four transplastomic plants when probed with *uidA* gene-specific sequences (Fig. 3C). Most importantly, the intensity of the band is comparable between all four transplastomic plants (Fig. 3C), unlike the transcription of *uidA* under the *psbC* promoter where transcript levels were significantly low when CUG was the start codon (Fig. 2C). Further, probing of the blot with 16S rRNA confirmed the equal loading of total RNA in all lanes (Fig. 3D).

### Expression of GUS under different start codons in transplastomic tobacco plants

**Figure 4:**
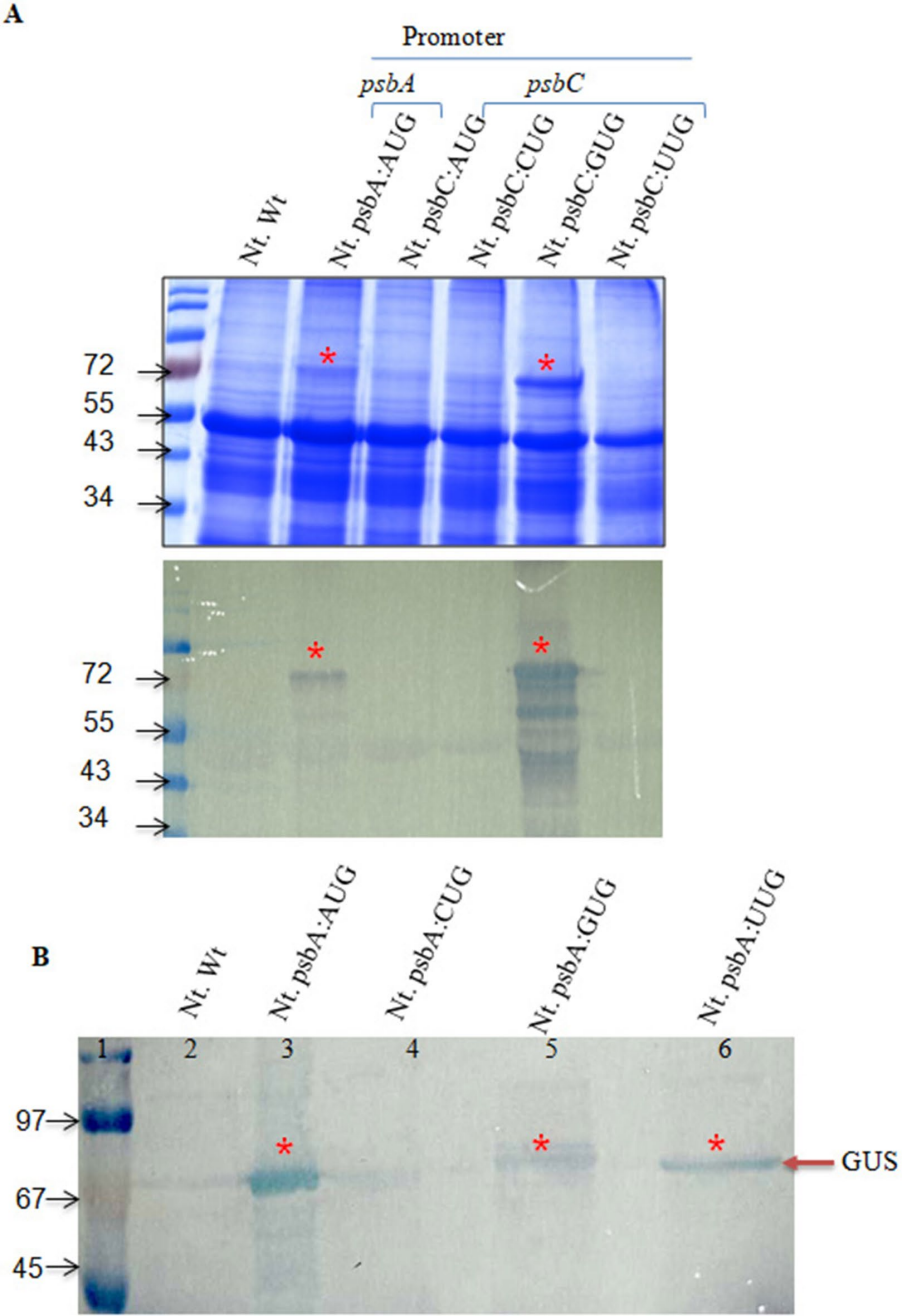
Comparative protein profile analyzed on (**A**) SDS-PAGE and corresponding western blot using anti-GUS antibodies from the transplastomic tobacco leaf. The details of promoter and start codon utilized for *uidA* expression are given just above the lane and wild type untransformed *Nicotiana tabaccum* (Nt. Wt) was used as a negative control. Note the high expression of GUS under psbC promoter having GUG as the start codon. (**B**) Western blot analysis of transplastomic tobacco plants expressing *uidA* with one of the four start codons (AUG, CUG, UUG, GUG) under psbA promoter. Asterisk symbol point out the expected size GUS protein band.

To assess the influence of the start codon on the translation efficiency, GUS expression under four-start codons (AUG, GUG, CUG, and UUG) driven by either *psbC* or *psbA* promoters was examined initially based on protein profile using SDS–PAGE (Fig. 4). A protein band corresponding to the expected size of GUS protein could be seen in the plants where *uidA* was placed under psbA:AUG, and psbC:GUG (Fig. 4A) when crude protein extract was loaded in the gel. These results were confirmed further using western blot analysis. Also, the presence of a GUS protein was observed in the transplastomic plants where AUG, GUG, and UUG start codons were utilized for *uidA* gene expression driven by *psbA* promoter (Fig. 4B). However, no GUS corresponding protein band was observed in the western blot in the transplastomic plants where CUG was the start codon. To confirm the presence of the GUS protein in the transplastomic plants transformed with psbA:AUG, psbA:GUG, psbA:UUG constructs, an immunoprecipitation experiment was carried out using anti-GUS antibodies. The expected size GUS band was observed in SDS-PAGE and western blot analysis with immunoprecipitated proteins from plants transformed with psbA:AUG, psbA:GUG, psbA:UUG constructs, while no corresponding band was found in the wild-type plant (Supplementary Fig. S4). Furthermore, the histochemical staining to detect β-glucuronidase (GUS) activity exhibited the presence of blue colur in transplastomic plants expressing these constructs (Supplementary Fig. S5).

### Comparison of GUS expression between *psbC* and *psbA* promoters in chloroplasts

**Figure 5:**
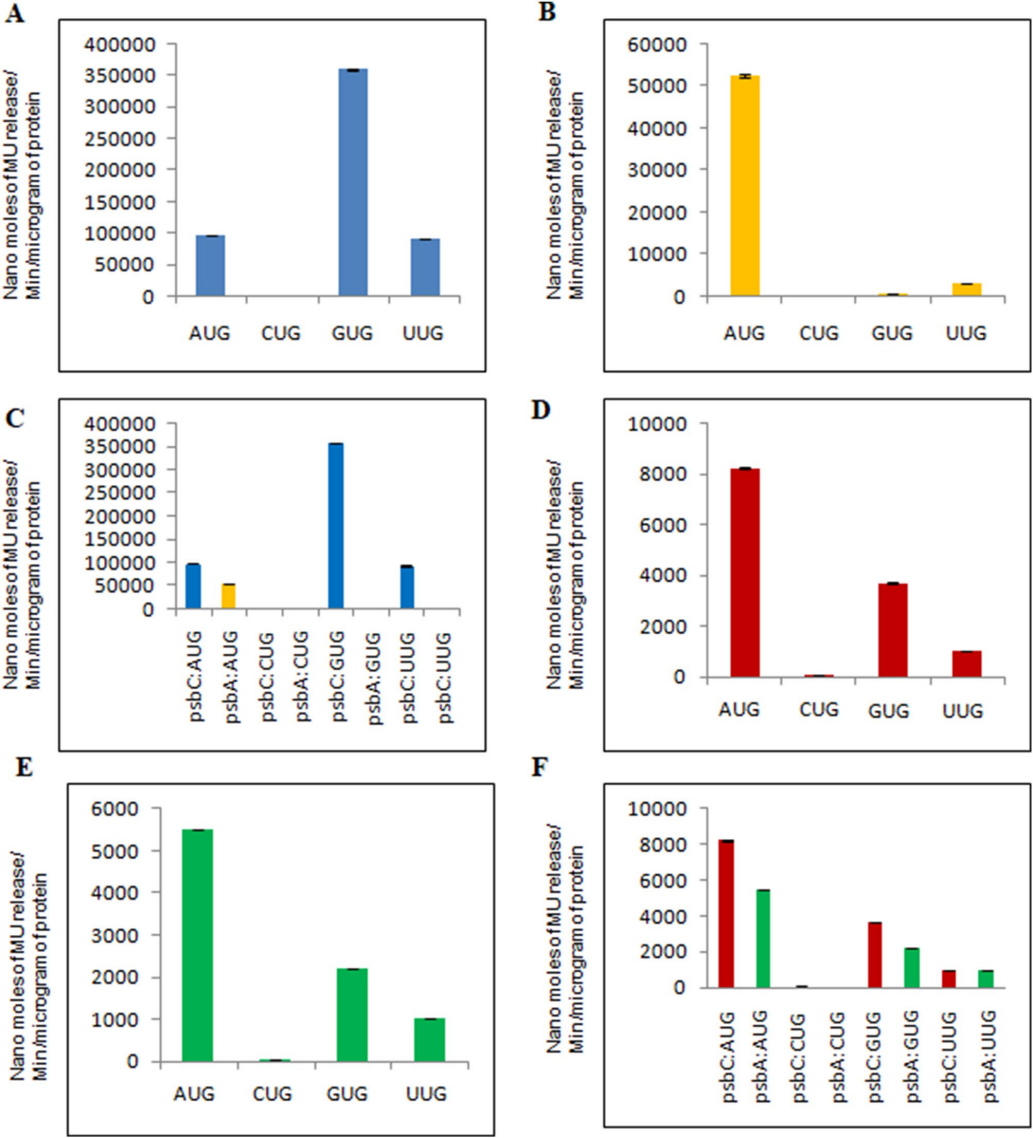
Comparison of GUS expression levels in transplastomic tobacco plants under different translation initiation codons (AUG, CUG, GUG, and UUG), driven by either tobacco *psbC* or rice *psbA* promoter and comparison of same constructs expressed in *E. coli*. Expression of GUS in tobacco chloroplasts under the regulation of native tobacco *psbC* promoter (**A**) and heterologous rice *psbA* promoter (**B**). Expression of GUS in *E. coli* under tobacco *psbC* promoter (**D**) and *rice psbA* promoter (**E**). Comparison of GUS expression under *psbA* and *psbC* promoters in *N. tabaccum* (**C**) and *E. coli* (**F**).

To compare the GUS expression between the transplastomic lines developed using eight different constructs, a quantitative GUS assay based on the enzymatic activity was performed. The GUS expression under four start codons driven by the *psbC* promoter exhibited highest GUS activity when GUG was the translation initiation/start codon, followed by AUG and UUG (Fig. 5A). It is worth noting here that GUG is the start codon for *psbC* in wild-type tobacco plants. No detectable GUS activity was observed when CUG was the start codon, which could be attributed to the lack of sufficient mRNA to translate or possible rapid degradation. Unlike GUS expression under the *psbC* promoter, its expression under the *psbA* promoter showed significant changes depending upon the start codon used. Among the four transplastomic lines, the highest GUS activity was observed when AUG was the start codon, which is also the canonical start codon of *psbA* in both rice and tobacco (Fig. 5B). Although GUS activity was detected under GUG and UUG start codons, the level of expression was very low (669 and 3210 nmol of MU release/minute/microgram of protein, respectively) as compared to its activity under the AUG start codon. Again, GUS activity could not be detected when CUG was the start codon, irrespective of the promoters (*psbA* or *psbC*) used, which is similar to the results obtained with the *psbC* promoter.

To delineate the role of non-AUG codons and the sequence context surrounding the translation initiation site in gene expression, GUS activity was compared between the *psbC* and *psbA* constructs having the same start codon but differed in their upstream and downstream sequences (Supplementary Figs. S2B and S6). The comparative GUS activity data showed that GUS expression was highest when *uidA* was expressed under the tobacco *psbC* promoter with GUG as the start codon, the native sequence under which *psbC* gene is expressed in wild-type tobacco plants (Fig. 5C). Interestingly, the level of GUS expression under the *psbC* promoter with the GUG start codon is more than double as compared to that of the GUS expression observed under the *psbA* promoter with AUG as the start codon (Fig. 5C).

### Comparison of GUS expression under *psbC* and *psbA* promoters in *E. coli*

As transcription and translation mechanisms of chloroplasts share significant similarities with bacterial systems due to the prokaryotic origin, and yet chloroplasts have evolved several additional features, we were interested to assess the impact of evolutionary changes in the transcription and translation systems on gene expression due to usage of different start codons and the TIS sequence context in bacteria. For this purpose, we transformed all eight constructs (four each of *psbC:uidA* and *psbA:uidA*) into *E. coli* and compared GUS activity with chloroplast expression. The GUS expression could be seen in *E. coli* under both *psbC* and *psbA* promoters. The expression profile of GUS under the same start codon is comparable between *psbC* and *psbA* promoters in *E. coli* (Fig. 5D,E). These results are in contrast to the GUS expression profile observed in chloroplasts where the expression profile of GUS changed significantly between *psbC* and *psbA* promoters (Fig. 5A,B). Interestingly, there was no detectable GUS activity when CUG was the start codon in *E. coli* as well, similar to the results obtained in chloroplasts.

### Comparison of GUS expression between chloroplasts and in *E. coli*

Significant differences between chloroplasts and *E. coli* in terms of GUS expression profile and levels of expression were observed depending on the start codon and the promoter combination used. While the highest GUS activity was observed in *E. coli* when AUG was the start codon, irrespective of the promoter used (Fig. 5D–F), its expression in chloroplasts was highest when *uidA* was expressed under the *psbC* promoter with GUG as the start codon (Fig. 5C). Again, significant differences were observed in the GUS activity when compared between those of chloroplasts and *E. coli* when UUG is used as the start codon (Fig. 5C,F). While the expression level of GUS with UUG as the start codon was similar in *E. coli*, its expression level in chloroplast was much higher under the *psbC* promoter as compared to the *psbA* promoter. The statistical analysis of the GUS enzyme activity based on three replicates showed that the GUS activity varied significantly depending on the start codon and the promoter combination used (*P* < 0.001).

### Non-AUG start codon tested was not subject to RNA editing in chloroplasts

**Figure 6:**
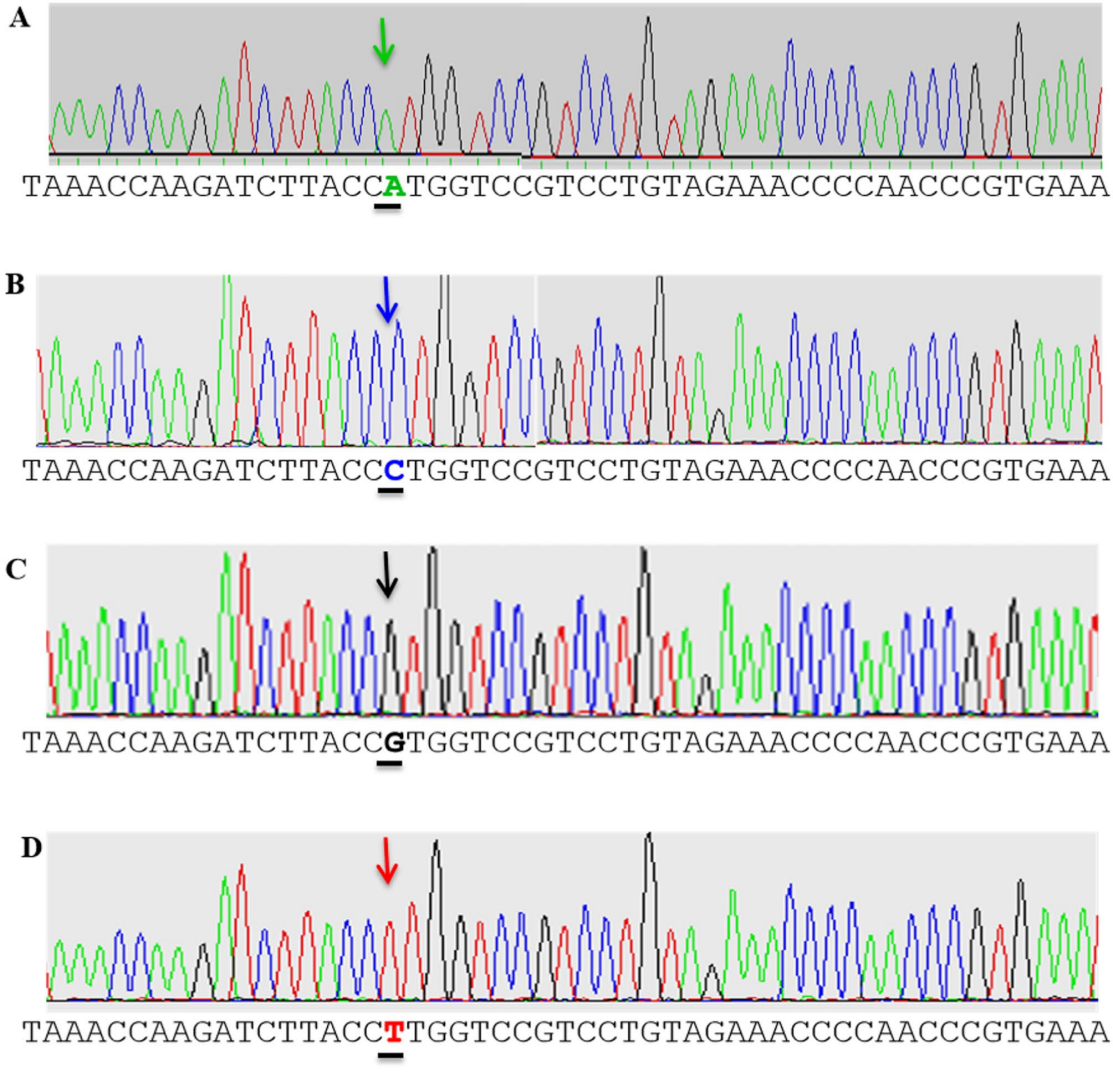
Partial sequence of *uidA* transcripts isolated from transplastomic plants that had different translation initiation start (TIS) codons. Transplastomic tobacco plants expressing psbA:*uidA* gene under AUG (**A**), CUG (**B**), GUG (**C**) and UUG (**D**) start codons, respectively. Sequencing data showed that the transcripts had the same base as per the gene construct in each of the transplastomic plants, ruling out any RNA editing of *uidA* transcripts in these plants.

As RNA editing is a common phenomenon in chloroplasts, especially since start codons are created by editing transcripts post-transcriptionally, we were interested to see whether the *uidA* transcripts with modified start codons are subject to RNA editing to revert to the native start codon before translation in the chloroplasts. To verify this, we analyzed the *uidA* transcripts expressed under the *psbA* promoter with different start codons and by sequencing respective cDNAs. Sequence analysis showed that there were no changes in the cDNA sequence in any of the four transplastomic plants when compared to the corresponding DNA sequence present in the vector used to transform the chloroplasts (Fig. 6). These results suggest that mRNA with mutations in the first nucleotide of the start codon tested in this study has not been subjected to RNA editing phenomenon present in the chloroplasts.

### Translation initiates from multiple sites in chloroplasts and it varies depending on the sequence context of TIS

**Figure 7:**
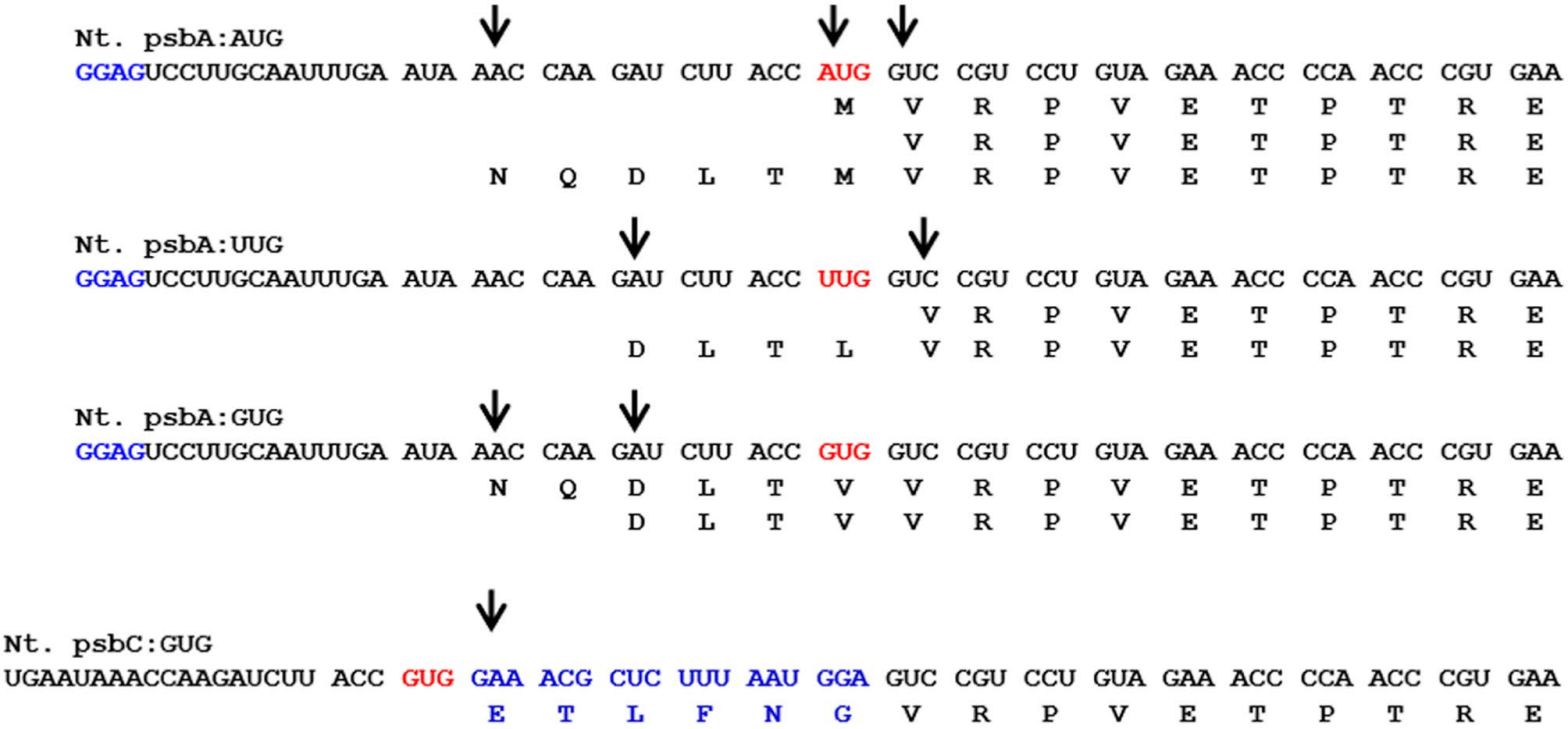
Translation initiation of GUS protein expressed under different start codons in transplastomic tobacco chloroplasts. Each arrow indicates the translation initiation site as determined by mass spectrometry-based peptide sequencing (MS/MS). Note that GUS protein is translated from multiple sites that are upstream to the expected start codon (shown in red) and even when the universal start codon (AUG) was present in the transcript downstream to the SD sequence. Note that whereas protein translation initiated from three and five upstream codons when GUS was expressed with the GUG start codon under the psbA promoter, it initiated from the single site when expressed under the psbC promoter, indicating the role of the sequence context besides the start codon.

Although the AUG start codon plays an important role in the initiation of protein translation, we were interested to see how tRNAs and ribosomes recognize and initiate protein synthesis when AUG is replaced with a non-AUG codon. The proteomics-based N-terminal sequence data revealed several interesting aspects of protein translation initiation and post-translational modifications in the chloroplasts. As can be seen from the summarized proteomics data (Fig. 7, Supplementary Table S1 and Supplementary Fig. S7), translation is initiated from multiple and, in some cases, novel sites that are upstream to the expected start codon. As can be seen from Fig. 7, translation is initiated from two or more sites, and the sites of initiation varied depending on the non-AUG start codon. In contrast, translation was initiated from a single site when *uidA* was expressed under the psbC promoter with GUG as the start codon, which is also the native start codon for psbC. Further, the N-terminal sequence of GUS revealed a number of post-translational modifications that included N-terminal methionine excision and N-terminal methionine formylation (Supplementary Table S1).

## Discussion

### Expression of *uidA* under tobacco *psbC* and rice *psbA* promoters with different start codons

A number of genes encoded by the chloroplast genome in terrestrial plants were shown to be translated from non-AUG start codons^32^. Conversion of AUG codon to AUU or AUC in chloroplast *petD* gene in the green alga *Chlamydomonas* was shown to reduce the translation initiation rate by 10–20% as compared to AUG^33^. On the other hand, the conversion of UUG, a non-AUG codon, to AUG was shown to enhance the translation efficiency of chloroplast *infA* gene in another green alga *Chlorella vulgaris* by 300-fold^22^. Further, the change of the AUG start codon to non-AUG codons was shown to influence the protein translation efficiency in *Chlamydomonas*^34^. For example, conversion of the AUG start codon of *petA* gene (encoding cytochrome f) to AUU, ACG, ACC, ACU, and UUC was shown to influence cytochrome-f levels in *Chlamydomonas* chloroplast significantly^34^. In addition, sequence downstream to the AUG start codon was also shown to influence the translation initiation and elongation of protein synthesis in tobacco chloroplasts^27^. The present study was undertaken to understand the role of the start codon and its flanking region in the gene regulation at transcription and translation stages in chloroplasts of higher plants. For this purpose, we chose the promoter of tobacco *psbC* gene having GUG as the native start codon^35^ and another promoter of rice *psbA* gene with AUG as the native start codon^30^ to express *uidA* (GUS) reporter gene under four different start codons (GUG, AUG, UUG and CUG) and assessed the role of start codons and surrounding sequence context in gene expression at transcription and translation in vivo in the stably transformed tobacco chloroplasts. The constructs having rice *psbA* promoter and terminators were used to compare gene expression under a different sequence context surrounding the start codon and, also to avoid any possible homologous recombinations between native and introduced promoter/terminator, if any other than the desired locus (rbcL-accD), that may, leading to large DNA deletions and rearrangements in the plastid genome^28^.

An important finding in the present study is the very low level of transcription of *uidA* under the *psbC* promoter with CUG as the start codon (Fig. 2C) as compared to other start codons (AUG, GUG or UUG) tested. On the other hand, there are no significant differences in the transcript levels of *uidA* when expressed under the *psbA* promoter irrespective of the start codon used (Fig. 3C). It is possible that the transcription factors that bind the promoter region and recruit RNA polymerase to initiate transcription are no longer able to recognize the promoter element, hence leading to the lack of efficient transcription of *uidA*. These results further emphasize the previous finding that the overall sequence context around the start codon is important than the start codon itself for efficient transcription of genes in chloroplasts^27^, similar to prokaryotes^36–38^ and eukaryotes^39^.

Another important finding in this study is the higher expression of GUS with GUG as the start codon than with the AUG start codon when expressed under the *psbC* promoter, while the expression is just reverse when expressed under the *psbA* promoter (Fig. 5A,B). Importantly, the expression level of GUS under the *psbC* promoter with AUG and UUG start codons is almost double compared to the highest expression level observed under the *psbA* promoter with AUG as the start codon (Fig. 5C), suggesting that the sequence context of the *psbC* promoter surrounding the translation initiation is the most favorable for protein translation in chloroplasts. However, the same sequence context is not favorable in *E. coli*, where the sequence context with AUG is the most favorable under both *psbA* and *psbC* promoters (Fig. 5D,E). The comparative expression data between chloroplasts and *E. coli* suggest the significance of not only the sequence context and start codons in protein translation efficiency but also the evolutionary changes that took place in the protein translation machinery of chloroplasts from where they evolved. It is possible that chloroplasts might have selected and enriched different mutations that have a cumulative effect on gene expression over a period of time to express certain genes like *psbC,* a chlorophyll-binding protein of PSII complex that undergoes light-mediated turnover during photosynthesis^40^, as another layer of gene regulation as observed in other organisms^41^ including bacteria^26,42–46^.

Such a selection process would have also been facilitated by the plasticity built in the translation mechanism of chloroplasts with the parallel evolution of their ribosome and/or factors interacting with TIS and near-cognate start codons with mismatches. This view is supported by the observation that a number of genes with important functions in the bacteria^42,47,48^ and certain viral proteins required for the rapid multiplication of viruses are translated from non-AUG start codons^49,50^.

CUG has been shown to function as the start codon in several eukaryotic organisms^20,21,51,52^ and the second most efficient start codon after the universal AUG codon^20^. In contrast, in prokaryotes, nearly ~ 80% of annotated genes initiate at AUG codons, ~ 12% at GUG, and ~ 8% at UUG, with variable incidences of AUU, CUG, and AUC across species^47^. For instance, in 69 model bacterial genomes, CUG has been reported as a start codon in 20 annotated open reading frames/ ORFs/genes (0.024% ORFs suggesting negligible translation initiation frequency and less efficient start codon)^26^. However, in the present study the lack of GUS expression in both chloroplasts and *E. coli* with CUG as the start codon, irrespective of the promoter used, suggests organism-specific preferences.

### Transcripts having non-AUG start codons are not subject to RNA editing in chloroplasts

RNA editing, a phenomenon present in chloroplasts that creates the start codon or changes the amino acid sequence in the proteins by base editing at selected positions in the transcript, has not been found in non-AUG start codons with an altered nucleotide at the first base. RNA editing is a significant feature of gene regulation in terrestrial plant chloroplasts, by which the mRNA sequence is selectively altered post-transcriptionally, which is not observed in the algal chloroplasts or the bacteria^53^. RNA editing is used either to create a start codon or to alter the amino acid in the protein sequence. For instance, an AUG start codon is shown to be created before translation in the mRNA of *rpl2*, *psbL* and *ndhD* genes^53–56 ^in chloroplast through the conversion of C to U through RNA editing. In our study, the sequencing results of cDNAs corresponding to *uidA* transcripts representing all four transplastomic lines (Fig. 6) did not reveal any changes related to RNA editing, suggesting that RNA editing did not recognize the first base edited start codon, possibly due to the high selectivity of RNA editing, which is limited to certain codons only in selected genes^57^.

### Change in start codon leads to shift in protein translation initiation site (TIS)

Identification of TIS when the start codon is altered is another objective of the present study. Studies by Sasaki and Nakashima^58^ showed that the translation of an insect viral RNA is initiated with glutamine, without the participation of the initiator methionine tRNA. The tRNA that reads the AUG codon in eubacteria also reads GUG and UUG codons and translates the codons as formylmethionine^59^. Determination of N-terminal ends of GUS expressed under four different start codons showed protein translation from different codons upstream to the typical start codon, resulting in a heterogeneous population (isoforms) of proteins differing in their N-terminal ends, which in turn might be allowing organisms to select a particular isoform with different turnover rates, localization and biological function. Protein synthesis from codons other than AUG and amino acids other than methionine has been reported earlier^20,26,60,61^. Also, the CUG codon was shown to code for serine in vivo in *Candida albicans* instead of leucine as predicted by the genetic code^62^. In addition, our data showed that N-terminal methionine got modified and/or processed similar to that in prokaryotic and eukaryotic organisms^21,63,64^, suggesting a high degree of conservation in post-translational modifications across organisms and organelles.

## Conclusion

The comparative GUS expression under four-start codons that differed in their first base (**A**UG, **U**UG, **G**UG and **C**UG) under *psbC* and *psbA* promoters in tobacco chloroplasts and its expression under the same combination of the translation initiation site and start codons in *E. coli* revealed that the chloroplasts of the present-day land plants have acquired an additional layer of regulatory feature as compared to prokaryotic progenitors during the long course of evolution. In addition, proteomics-based N-terminal sequencing of GUS expressed in chloroplasts showed that protein translation initiates from multiple novel sites depending on the start codon used and TIS flanking it, again revealing that chloroplast has evolved enough plasticity that allows them not only to use non-AUG start codon to express particular gene but also continue to select and enrich a combination of mutations as a post-transcriptional regulatory mechanism to attain the required levels of proteins that undergo light-mediated rapid turnover during photosynthesis.

## Methods

### Vector construction, chloroplast transformation, and molecular and GUS assays

The plastid transformation vector pVSR326 (Fig. 1, GenBank Acc. No. AF527485, 28) was used to construct four vectors to express *uidA* under the *psbC* promoter (ppsbC:AUG, ppsbC:CUG, ppsbC:GUG and ppsbC:UUG) and another four vectors to express *uidA* under the *psbA* promoter (ppsbA:AUG, ppsbA:CUG, ppsbA:GUG and ppsbA:UUG) (Supplementary Fig S1). Note that in transcript/RNA Uracil (U) is used while in DNA/construct nucleotide Thymine (T) is used in place of Uracil. For consistency in terminology in figures and text, we have used the transcript nomenclature throughout the manuscript i.e. U (not T). These plastid transformation vectors are designed to contain flanking sequences (FLK) on either side of the transgene cassettes, derived from the host plant chloroplast genome, to facilitate two homologous recombinations for site-specific integration of transgenes. The vectors used in this study contained partial sequences *rbcL* and *accD* genes as flanking sequences for homologous recombination that integrate *uidA* (encoding ß-Glucuronidase) and *aadA* gene (encoding aminoglycoside adenine transferase) cassettes in the non-coding region present between *rbcL* and *accD* genes^65^. A three-step PCR-based procedure was followed to generate four psbC:uidA chimeric genes that differed in the first base of the start codon. In the first step, the *psbC* promoter of tobacco was amplified using the oligonucleotides psbC-SalI and psbCGUS-ATG/psbCGUS-CTG/psbCGUS-GTG/psbCGUS-TTG (Supplementary Table S2) and *Nicotiana tabcum* (tobacco) total genomic DNA as the template in 25 µl reaction volume for 30 cycles. In the second step, 5 µl of PCR mix from the first step was added to the second PCR mix that contained pVSR326^30^ plasmid DNA as the template, without any additional primers and amplified for 10 cycles. In the third step, 5 µl of PCR mix from the second step was used as the template in the third PCR mix that contained psbC-SalI and GUS3-SacI primers. The PCR-amplified psbC:uidA was digested with SalI and SacI enzymes and cloned into pVSR326 (GenBank Acc. No.AF527485.1) at the same sites to create ppsbC:AUG, ppsbC:CUG, ppsbC:GUG and ppsbC:UUG vectors.

The ppsbA:CUG, ppsbA:GUG and ppsbA:UUG vectors were generated using QuikchangeH Site-directed Mutagenesis Kit (Stratagene, La Jolla, CA) according to the manufacturer’s instructions. Primer pairs used in the mutagenesis are listed in Supplementary Table S2. Chloroplast transformation vector pVSR326, where *uidA* is placed under the *psbA* promoter of rice with the AUG start codon, was used to compare GUS expression under the *psbA* promoter with CUG/GUG and UUG start codons. All introduced mutations were verified by sequencing DNA.

All vectors contained rrn:aadA:rbcL (*aadA* gene driven by *rrn* promoter and *rbcL* terminator) selectable gene cassette imparting resistance to spectinomycin antibiotic. The tobacco plastid DNA sequences spanning *rbcL*:*accD* genes (nucleotides 58,056–60,627; EMBL, Z00044) were used for site-specific integration of transgenes into the plastome. Standard procedures were followed for cloning, Southern, Northern, and protein analysis^66^. A method based on the particle delivery system (Gene Gun method) was followed for tobacco (*Nicotiana tabacum* cv. Petit Havana) chloroplast transformation^65^. All eight constructs were also transformed into β-glucuronidase-deficient *E.coli* strain GMS407 using the method of Sambrook et al.^66^.

### GUS activity and histochemical assays

GUS activity assay and histochemical staining of β-glucuronidase (GUS) expression was performed as described elsewhere^67^. Briefly, for the GUS assay, about 100 mg transplastomic tobacco leaf tissue was homogenized in 300 µl of GUS extraction buffer containing 50 mM NaH_2_PO_4_, pH 7.0, 10 mM EDTA, 10 mM beta-mercaptoethanol followed by centrifugation at 10,000 rpm for 10 min. In the case of *E. coli* GMS407, cells were sonicated using the same GUS extraction buffer followed by separating the extracts using a centrifuge. The protein concentration in the extracts was estimated using the Bradford method^68^. A 4 µg of total protein was added to GUS assay buffer containing 1 mM 4-methyl umbelliferyl beta-d-glucuronide (MUG; Sigma M-9130), the total volume of extraction buffer was adjusted to 200 µl and incubated at 37 °C. After 10 min of reaction, 50 µl aliquot was taken and added to 950 µl stop buffer (0.2 M Na_2_CO_3_) to terminate the reaction. The amount of 4-methyl umbelliferone released was estimated by measuring the fluorescence emission at 360 nm excitation and 460 nm emission using the spectrofluorimeter. The fluorimeter was calibrated with freshly prepared 1 µM, 10 µM, and 100 µM methyl umbelliferone (MU) standards. GUS activity was calculated as Nano moles of MU release/minute/microgram protein. The level of nanomoles of MU released directly correlates with the level of GUS expression. The statistical analysis for the GUS activity assay was done using SAS statistical software version 9.3 (https://sscnars.icar.gov.in/) to see the level of significance. Standard errors and standard deviation were calculated from the replicates.

For histochemical GUS assay, 10 days old seedlings and leaf discs from 5-week-old transplastomic lines were incubated in 1 mM X-Gluc substrate in 50 mM NaH_2_PO_4_, pH 7.0 at 37 °C for 12–16 h. Photographs were taken under the binocular microscope after rinsing the seedlings/leaf disc 3 times in 70% ethanol for 5 min each. GUS expression was categorized based on the intensity of the blue color (low, medium, and high) in the leaf discs/seedlings observed under the microscope.

### cDNA synthesis and sequencing

The total RNA from psbA:AUG, psbA:CUG psbA:GUG, and psbA:UUG construct expressing transplastomic plants leaves were isolated using TRIzol reagent (Invitrogen, Life Technologies, USA) as per manufacturer’s instructions. The quality and quantity of total RNA were assessed by NanoDrop 1000 spectrophotometer. The RNA samples with A260/A280 ratio of ~ 2.0 and A260/A230 ratio of 2.0–2.2 were considered pure. The total RNA was stored at − 70 °C. The DNase-treated 1 µg total RNA was used for the synthesis of first strand cDNA using AffinityScript qRT-PCR cDNA synthesis kit (Stratagene, Agilent Technologies, USA) according to the manufacturer’s instructions. PCR amplification was performed in BIORAD thermal cycler using 25 µl of total volume reaction containing 100 ng template cDNA, 1X Taq Polymerase buffer, 1.5 mM magnesium chloride, 1 mM dNTPs, 0.4 µM of each forward and reverse primer pair and 2U of Taq DNA polymerase. At least two cDNAs were sequenced per line from Macrogen Corporation, South Korea.

### Protein extraction and SDS–PAGE analysis

Total protein from 0.5 g tobacco transplastomic plant leaf was extracted with 500 µl 2X PBS (pH 7.4) containing 0.1% Tween 20 and proteinase inhibitor cocktail (Roche) by grinding using mortar and pestle. The supernatant was obtained after centrifugation at 10,000 rpm for 10 min. The concentration of the protein in the supernatant was estimated by the Bradford method^68^. The protein samples were subjected to 12% sodium dodecyl sulphate–polyacrylamide gel electrophoresis (SDS–PAGE) separation^69^. The gels were either stained with Coomassie Blue to visualize the proteins or used for Western blotting. For psbA:AUG 20 ug total protein while for remaining samples 50 ug total protein was taken for SDS-PAGE experiment. Similarly, total protein from overnight grown untransformed *E. coli* GMS407 (negative control), a mutant strain of beta-D-glucuronidase, and transformed *E. coli* GMS407 cells expressing GUS (using different translation initiation codons such as AUG, CUG, GUG and UUG) either under tobacco *psbC* promoter or rice *psbA* promoter were extracted by breaking cells via sonication and subjected to SDS-PAGE analysis.

### Immunoprecipitation

Protein A Sepharose 4 Fast Flow beads (GE Healthcare) were washed thrice at 1200 rpm with 1X PBS. 10 µl rabbit anti-GUS antibody (Sigma Aldrich, catalog number G5420) was added in 200 µl washed protein A beads in 1X PBS and allowed to bound at 4 °C for 4 h under shaking. The antibody-protein A bead complex was washed thrice with 1X PBS. The total protein from the tobacco transplastomic plant leaf was added to the antibody-protein A complex and incubated in a cold room overnight under shaking. Three washes were performed with 1X PBS at 1200 rpm for 5 min. The antigen (GUS protein)-antibody-protein A bead complex was resuspended in 15 µl 4X SDS PAGE sample buffer by boiling. After immunoprecipitation, the samples were separated on 10% SDS–PAGE mini gels as described in the instruction manual for the Mini-Protean III Electrophoresis Cell (BioRad).

### Western blot analysis

After separating proteins using SDS-PAGE analysis, proteins were transferred/blotted onto a polyvinylidene difluoride (PVDF) membrane (Millipore) to facilitate antibody probing. The PVDF membrane was blocked in 4% BSA (bovine serum albumin) in TBS-Tween 20 (TBST) for 30 min, followed by washing thrice in TBST for 10 min. The membrane was incubated with rabbit-raised anti-GUS antibody (1:10,000 dilution) in TBST for 1 h. The membrane was then washed thrice for 10 min each in TBST and incubated with an anti-rabbit alkaline phosphatase-conjugated secondary antibody (1:10,000 dilution) (Sigma) for 30 min. The membrane was washed thrice for 10 min each and developed with Western Blue^®^ Stabilized Substrate for alkaline phosphatase (Promega). Please note that the lack of protein detection in western blot analysis despite high GUS enzyme activity in psbC:AUG and psbC:UUG expressing plants could be because the anti-GUS antibody somehow did not recognize GUS translated from these two constructs as the presence of different forms of GUS protein is possible due to initiation from multiple sites. Perhaps the majority of proteins in these two cases are initiated at some other sites downstream/upstream to UUG or AUG.

### In-gel digestion of proteins

After electrophoresis, the gel was stained with Coomassie Brilliant Blue. Protein bands were excised, chopped into small pieces, and destained with 200 µl 50% methanol/10% acetic acid. The gel pieces were then rinsed with autoclaved MilliQ water and equilibrated with 100 mM ammonium bicarbonate (NH_4_HCO_3_) for 15 min at room temperature with gentle agitation. The gel pieces were washed with 200 µl 1:1 (v/v) NH_4_HCO_3_ and acetonitrile, dehydrated with 100% acetonitrile for 15 min, and then vacuum-dried. The sample was subjected to reduction with 10 mM dithiothreitol (DTT) in 25 mM NH_4_HCO_3_ for 45 min at 56 °C followed by alkylation with 50 mM iodoacetamide (IAA) in 25 mM NH_4_HCO_3_ for 30 min in the dark at room temperature. The gel pieces were rinsed briefly with 1:1 NH_4_HCO_3_ and acetonitrile solution, dehydrated with 100% acetonitrile for 15 min, dried, and digested with 50 µl enzyme solution containing 15 ng/μl Asparagine-N (Cat no. V1621, Promega) or Glu-C (Cat no. V165A, Promega) or trypsin in 25 mM NH_4_HCO_3_ for 18 to 20 h at 37 °C. Following digestion, peptides were extracted twice with 100 μl of 1% trifluoroacetic acid in 60% acetonitrile, and the extracts were concentrated under a vacuum. The peptide pellet was stored at − 80 °C until further use.

### Nano-LC-based reverse-phase separation

Peptide pellets were redissolved in 20 μl of 0.1% TFA, and 15 μl of the sample was bound onto a 100 μmi.d. × 20 mm Easy-nLC precolumn (Proxeon Biosystems) at a flow rate of 5 μl/min. Reverse-phase separation of the peptide mixture was performed in a nano-LC system (Proxeon Biosystems) using a C-18 analytical column of 75 μmi.d. X 100 mm length at a flow rate of 300 nL/min with a solvent system comprising 0.1% TFA in 5% ACN , v/v (solvent A) and 0.1% TFA in 90% ACN (solvent B). The gradient was 0% B for 5 min, followed by a linear gradient of 0% to 45% B for 65 min, 45% to 100%B for 1 min, and 100%B for 10 min. The column eluates were directed to a Proteineer fc fraction collector (Bruker Daltonics), and fractions were spotted every 10 s onto a pre-spotted anchor chip 384/96 (PAC) target plates.

### Protein identification from 1D gel phase digested samples

Peptide samples were injected into a nano-LC system, fractionated, and spotted onto PAC (Pre-Anchored Chip) targets^70^. Sample plates were subjected to MADLI TOF/TOF-based acquisition in the automated mode through WARP-LC 1.2 (Workflow Administration by Result driven Processing, Bruker Daltonics) software tool. The MALDI TOF/TOF instrument parameters mentioned in Kumar et al.^70^ were employed for automatic acquisition: carrier plates with samples were subjected to pre-teaching to ensure the optimum and complete acquisition of 448 sample spots. Mass list calculation was done through the WARP-LC interface. A manually updated background list containing trypsin autolysis peaks, matrix peaks, and keratin peaks was used during mass list generation. Spectral peaks (*m/z*) corresponding to background peaks were excluded for MS/MS measurement. Only those peaks (precursors) that have an S/N > 20 were included in the measurement. Post-acquisition processes including mass annotation, baseline subtraction, and smoothening were performed using Flex Analysis software, version 3.0, through WARP-LC. Protein identification was achieved using Biotools, version 3.2, through an in-house-licensed Mascot server (version 2.3, March 2010).

## Supplementary Information

Supplementary Information 1.

Supplementary Information 2.

Supplementary Information 3.

Supplementary Information 4.
