# Supplementary material for: Chloroplasts evolved an additional layer of translational regulation based on non-AUG start codons for proteins with different turnover rates
Source: Sci Rep. 2023 Jan 17;13:896. doi: 10.1038/s41598-022-27347-9 (PMC9845219; doi:10.1038/s41598-022-27347-9)
Supplement: Supplementary file 1 — Supplementary Information 1. [file 41598_2022_27347_MOESM1_ESM.pdf]

Original, full length and unprocessed version of images

Figure 2 A

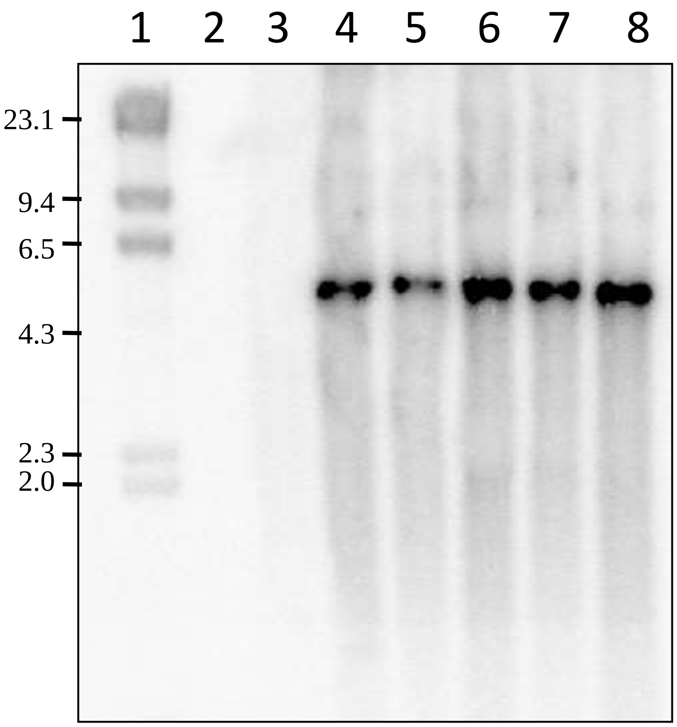

uidA Probe

Figure 2 B

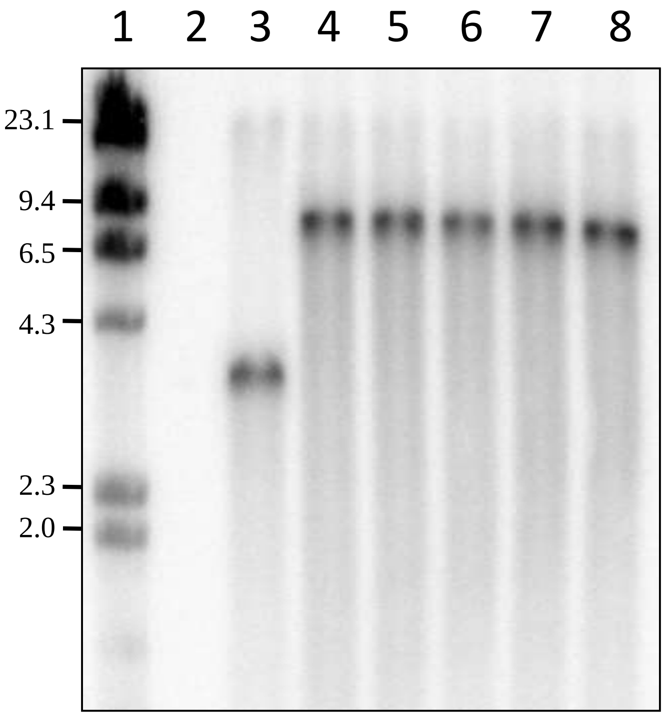

rbcL:accD Probe

Lane 1-8 represent **:**

1. Lamda DNA HindIII cut Marker
2. Blank
3. Nt. Wt (untransformed wild type *Nicotiana tabaccum*)
4. Nt.psbC:AUG transplastomic plants
5. Nt.psbC:CUG transplastomic plants
6. Nt.psbC:UUG transplastomic plants
7. Nt.psbC:GUG transplastomic plants
8. Nt.psbA:AUG transplastomic plants

Stable integration of *uidA* transgene in tobacco chloroplasts under tobacco *psbC* promoter with four different start codons. Southern hybridization of total genomic DNA probed with *uidA* (**A**) and partial gene sequences of *rbcL-accD* (**B**). Note that in transcript/RNA Uracil (U) is used while in DNA/construct nucleotide Thymine (T) is used in place of Uracil. For consistency in terminology, we have used the transcript nomenclature throughout i.e. U (not T).

Figure 2 C

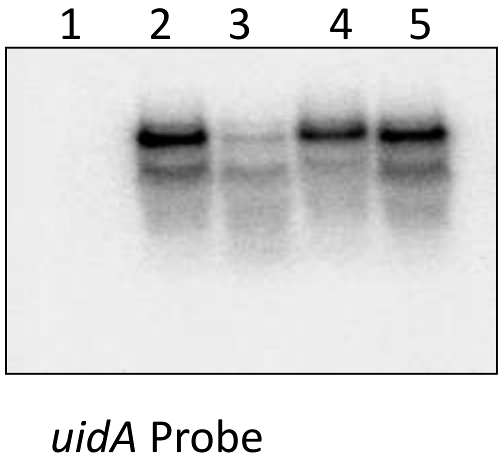

Figure 2 D

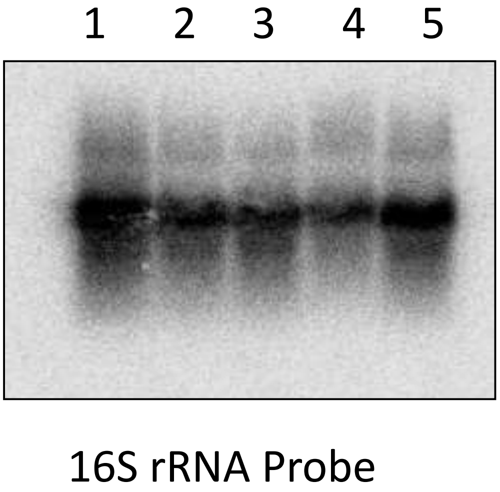

Lanes 1-5 represent :

1. Nt. Wt (untransformed wild type *Nicotiana tabaccum*)
2. Nt.psbC:AUG transplastomic plants
3. Nt.psbC:CUG transplastomic plants
4. Nt.psbC:UUG transplastomic plants
5. Nt.psbC:GUG transplastomic plants , respectively.

Expression analysis of *uidA* gene in tobacco chloroplasts under the tobacco psbC promoter with four different start codons. **(C)** Northern hybridization showing transcription of chimeric *uidA* under four different start codons in tobacco transplastomic plants. **(D)** The same blot reprobed with 16S rRNA to show equal loading of total RNA.

Figure 3 A **Figure 3 B**

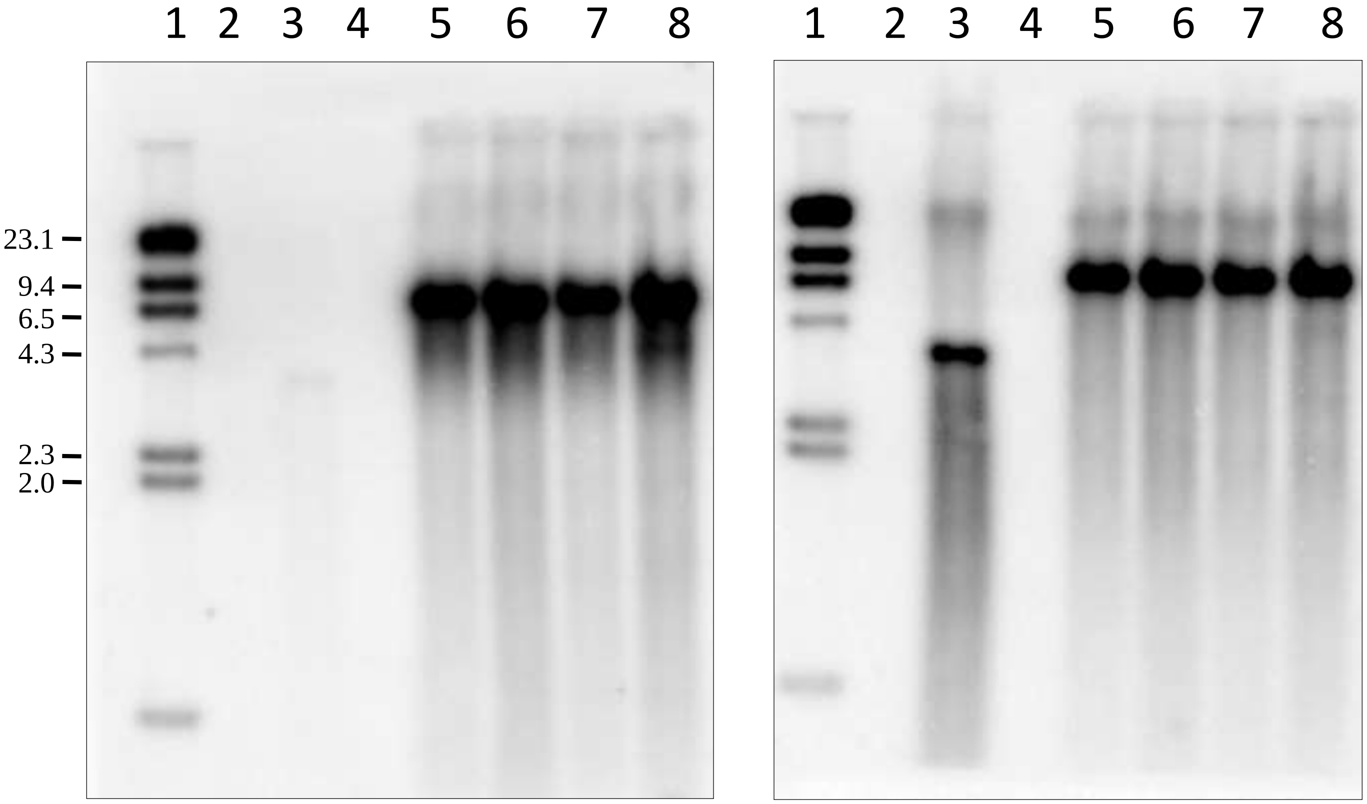

uidA Probe

rbcL:accD Probe

Lane

1. Lamda DNA HindIII cut Marker
2. Blank
3. Nt. Wt (untransformed wild type *Nicotiana tabaccum*)
4. Blank
5. Nt.psbA:CUG
6. Nt.psbA:UUG
7. Nt.psbA:GUG
8. Nt.psbA:AUG

Southern hybridization of genomic DNA using *uidA* **(A)** and partial *rbcL:accD* **(B)** gene sequences to show the site-specific integration of transgenes into tobacco plastome.

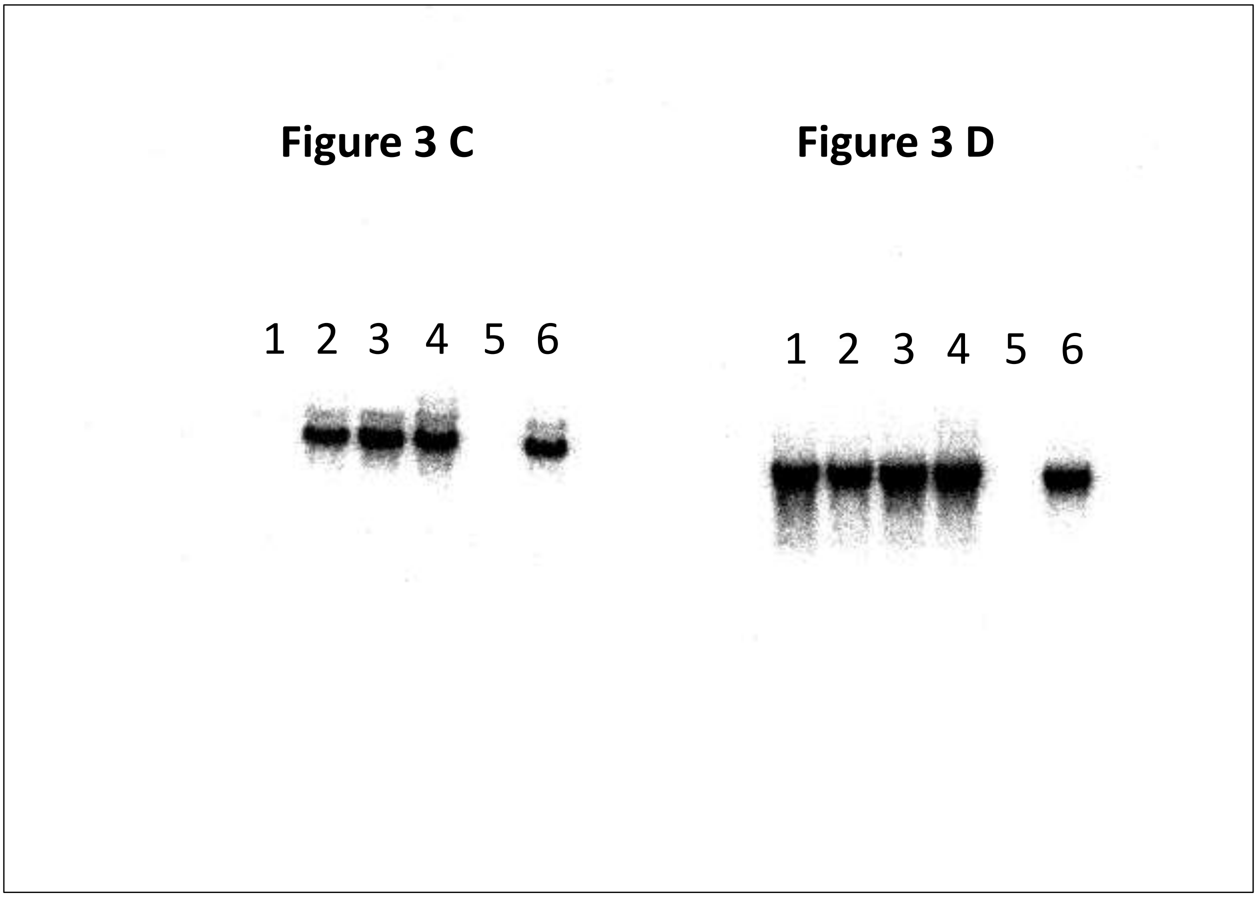

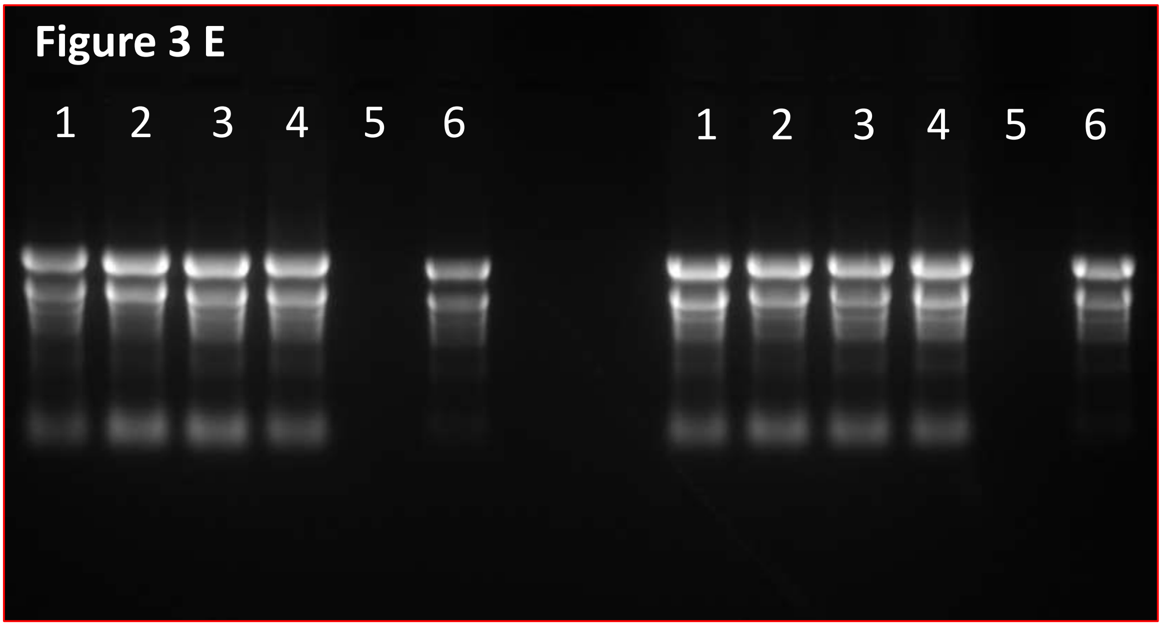

Northern hybridization showing the transcription of chimeric *uidA* in four different transplastomic plants **(C)** and the blot probed with 16S rRNA to show loading of total RNA **(D)**. **(E)** Agarose gel showing equal amount of RNA (10 µg) loading in lanes 1- 4 while half (5µg) was used for lane 6. Lanes 1-6 represent Nt. Wt (untransformed wild type *Nicotiana tabaccum*), Nt.psbA:CUG, Nt.psbA:UUG, Nt.psbA:GUG, blank and Nt.psbA:AUG transplastomic plants , respectively.

Figure 4 A

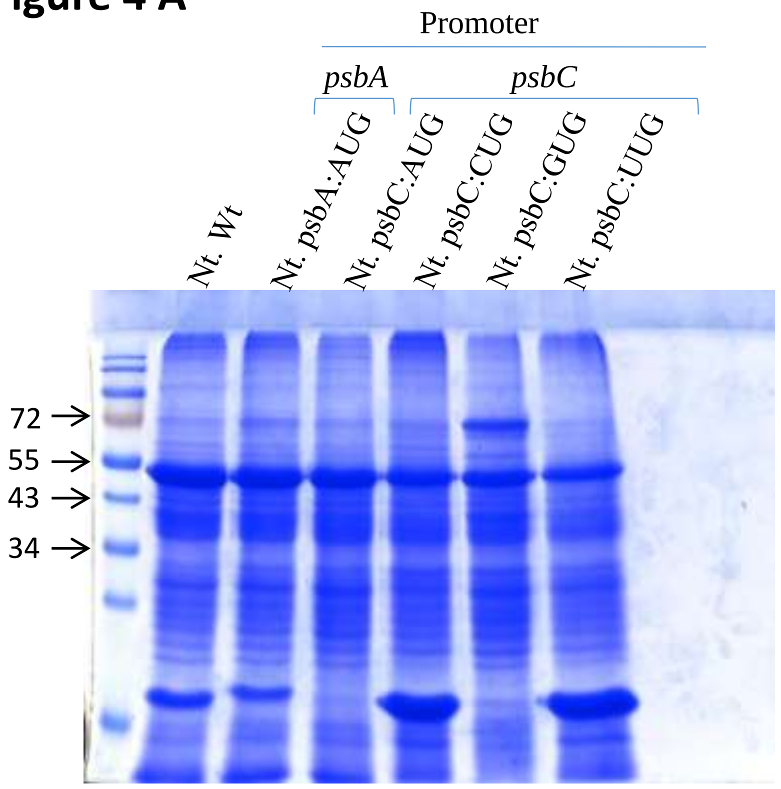

Comparative protein profile analyzed on 12 % SDS-PAGE using 50 ug total protein.

Figure S4

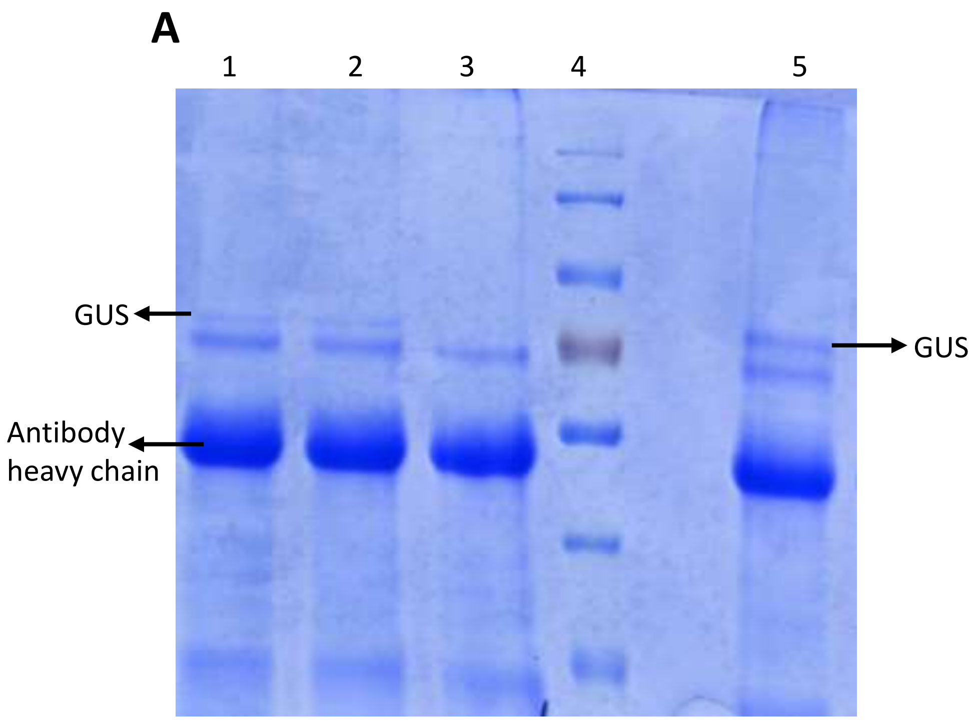

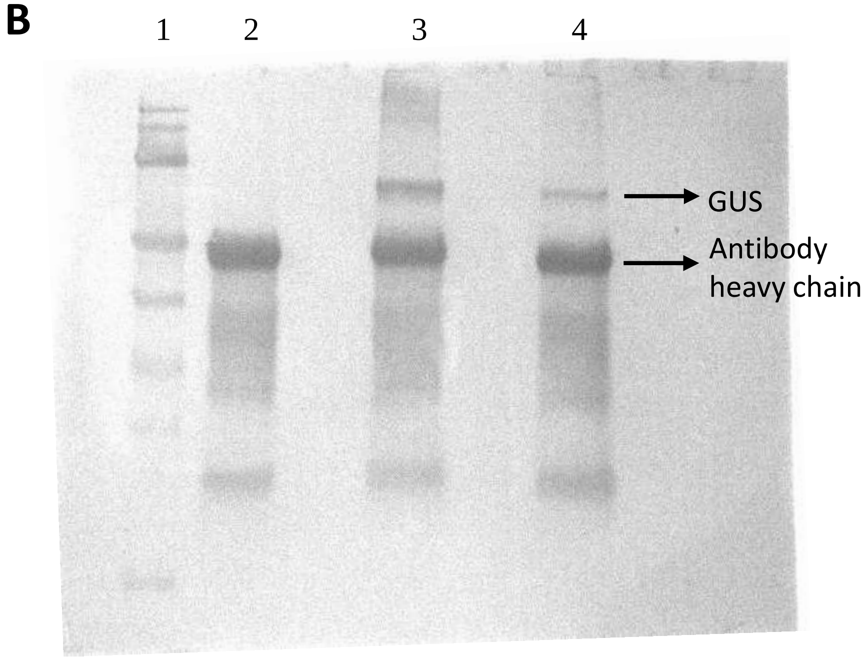

Figure S4. (A). SDS-PAGE analysis of immunoprecipitated proteins using anti-GUS antibodies from the transplastomic tobacco leaf total protein. Arrows and asterisk symbols point out the expected size GUS protein band. Lane 1-5 represents psbA:UUG, psbA:GUG, negative control (wild type/untransformed), protein ladder (in kilodalton/kD) and psbA:AUG construct expressing plants, respectively. **(B)**. Western blot analysis of immunoprecipitated proteins recognized by anti-GUS antibody from transplastomic tobacco leaf protein. Lane 1-4 corresponds to protein ladder (in kilodalton/kD), negative control (wild type), psbA:AUG and psbA:UUG construct expressing plants, respectively.
