# Supplementary material for: Chloroplasts evolved an additional layer of translational regulation based on non-AUG start codons for proteins with different turnover rates
Source: Sci Rep. 2023 Jan 17;13:896. doi: 10.1038/s41598-022-27347-9 (PMC9845219; doi:10.1038/s41598-022-27347-9)
Supplement: Supplementary file 4 — Supplementary Information 4. [file 41598_2022_27347_MOESM4_ESM.pdf]

SUPPLEMENTARY DATA

Chloroplasts evolved an additional layer of translational regulation based on non-AUG start codons for proteins with different turnover rates

Leelavathi Sadhu[1*], Krishan Kumar[1,2*], Saravanan Kumar[1,3], Abhishek Dass[1], Ranjana Pathak[1,4], Amit Bhardwaj[1,5], Pankaj Pandey[1], Nguyen Van Cuu[1,6], Bhupender S. Rawat[1] and Vanga Siva Reddy[1*]

1. Plant Transformation Group, International Centre for Genetic Engineering and Biotechnology (ICGEB), New Delhi, 110067, India.
2. Krishan Kumar, ICAR-Indian Institute of Maize Research, Delhi Unit, Pusa Campus, New Delhi, 110012, India.
3. Saravanan Kumar, Proteomics facility, Thermo Fisher Scientific, Bangalore, Karnataka, 560066, India.
4. Ranjana Pathak, Department of Microbiology & Immunology, Centre for Infectious Diseases, Stony Brook University, Stony Brook, New York 11794, USA.
5. Amit Bhardwaj, Perlmutter Cancer Center, NYU Langone Health, NYU Langone Medical Center, New York 10016, USA.
6. Department of plant pathology, Institute of Agricultural Genetics (AGI), Hanoi, Vietnam.

[*]To whom correspondence should be addressed. LS: Email:leelavathisadhu@gmail.com; KK: Email: krishan.kumar6@icar.gov.in; VSR: Email: vsreddy@gmail.com

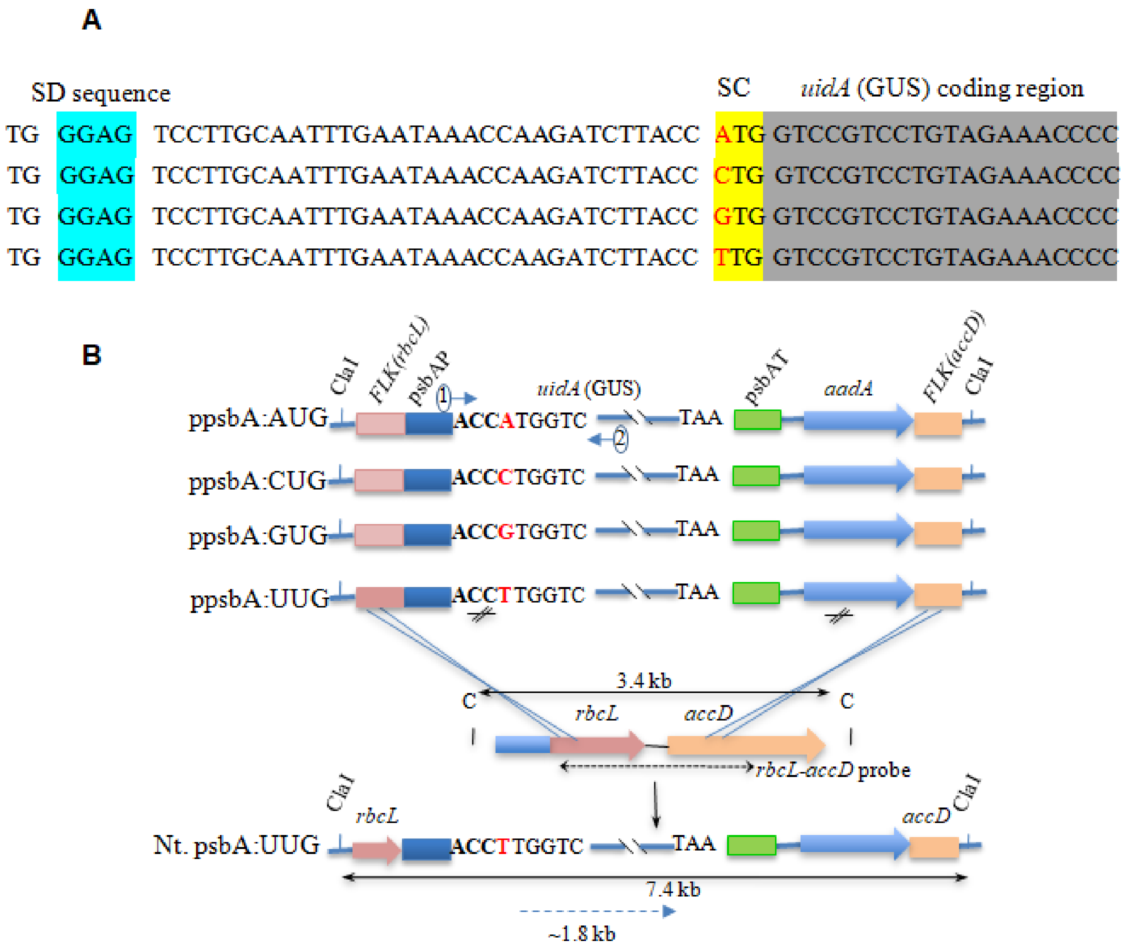

Supplementary Fig S1. **(A).** Partial sequence of *uidA* (GUS) gene fused-in-frame with *psbA* gene 5' UTR. SD: Shine-Dalgarno Sequence and SC: Start codon highlighted in blue and yellow, respectively. **(B)**. Partial restriction map showing the *uidA* and *aadA* genes. Also shown is the site of wild-type tobacco plastome where the *uidA* and *aadA* genes are expected to be integrated into the chloroplast genome and the resulting transplastome with restriction sites and anticipated size of DNA figments when restricted with ClaI.

Note that in transcript/RNA Uracil (U) is used while in DNA/construct nucleotide Thymine (T) is used in place of Uracil. For consistency in terminology, we have used the transcript nomenclature throughout the manuscript i.e. U (not T).

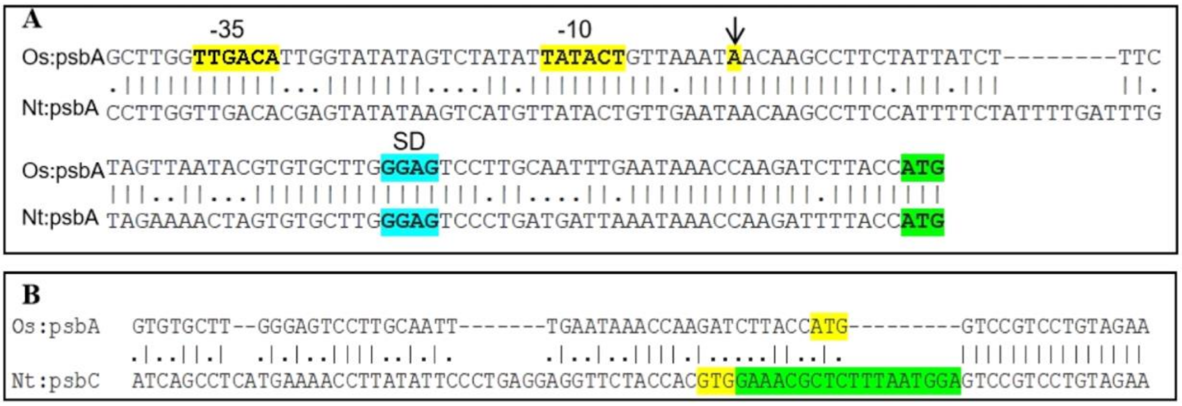

Supplementary Fig S2. (A) Comparison of partial *psbA* promoter region between *O. sativa* and *N. tabaccum*. **(B)** Comparison of sequences surrounding protein translation initiation codon of rice (*O. sativa*) *psbA* gene and tobacco (*N. tabaccum*) *psbC* gene.

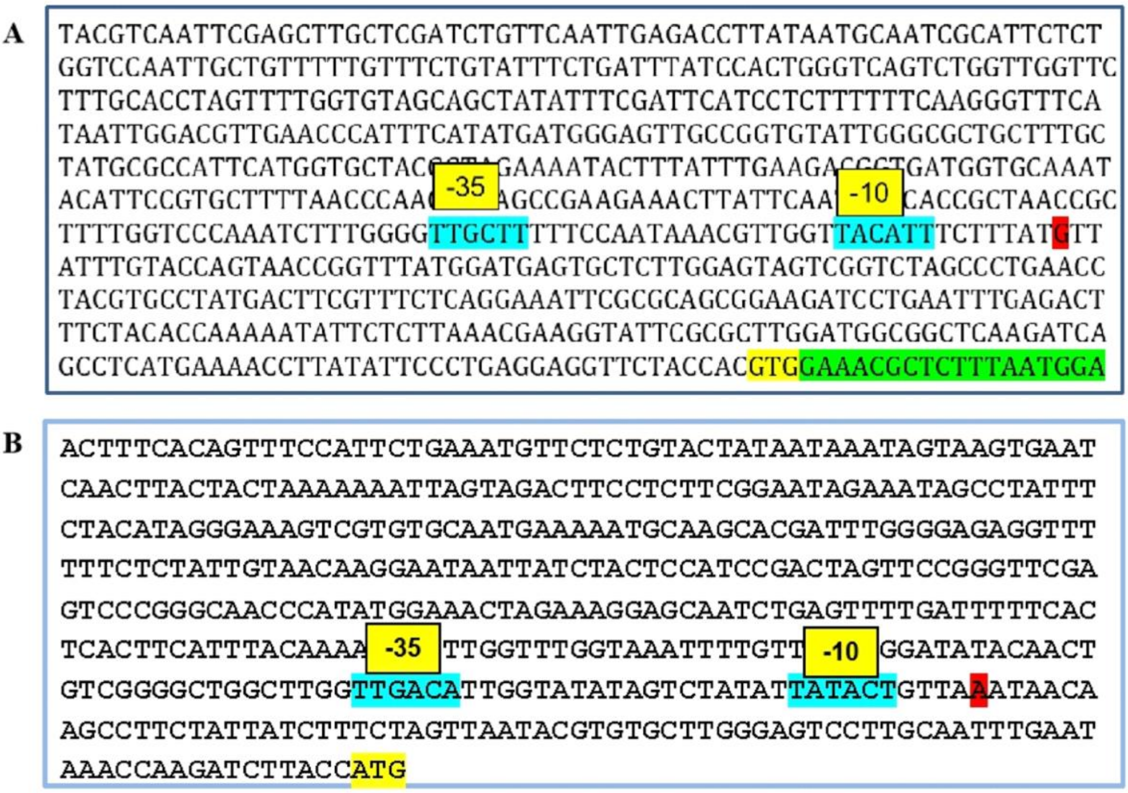

Supplementary Fig S3. Tobacco *psbC* promoter **(A)** and rice *psbA* promoter **(B)** sequences used to express *uidA* gene in the present study. The -35 and -10 elements are highlighted in blue. Also shown transcript initiation site (red) and start codon (yellow), 5' UTR and partial coding region. Note that the six N-terminal codons (green colored) of the *psbC* gene were fused in frame with *uidA* (GUS) to conserve the translation start site and translation initiation.

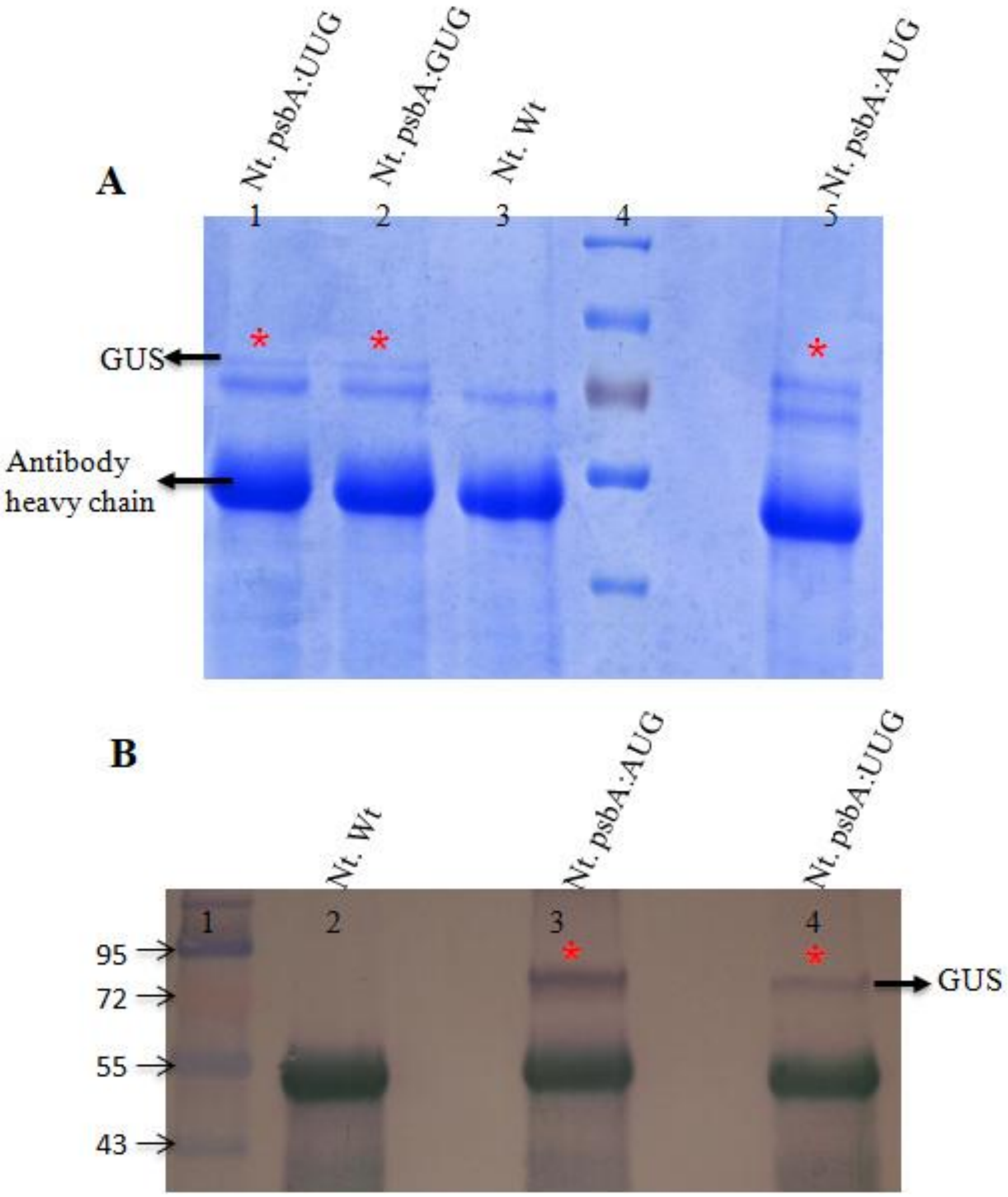

Figure S4. **(A)**. SDS-PAGE analysis of immunoprecipitated proteins using anti-GUS antibodies from the transplastomic tobacco leaf total protein. Arrows and asterisk symbols point out the expected size GUS protein band. Lane 1-5 represents psbA:UUG, psbA:GUG, negative control (wild type/untransformed), protein ladder (in kilodalton/kD) and psbA:AUG construct expressing plants, respectively. **(B).** Western blot analysis of immunoprecipitated proteins recognized by anti-GUS antibody from transplastomic tobacco leaf protein. Lane 1-4 corresponds to protein ladder (in kilodalton/kD), negative control (wild type), psbA:AUG and psbA:UUG construct expressing plants, respectively. Note the expression of GUS (encoded by *uidA* gene) driven by the psbA promoter having either AUG, UUG, or GUG as the start codon.

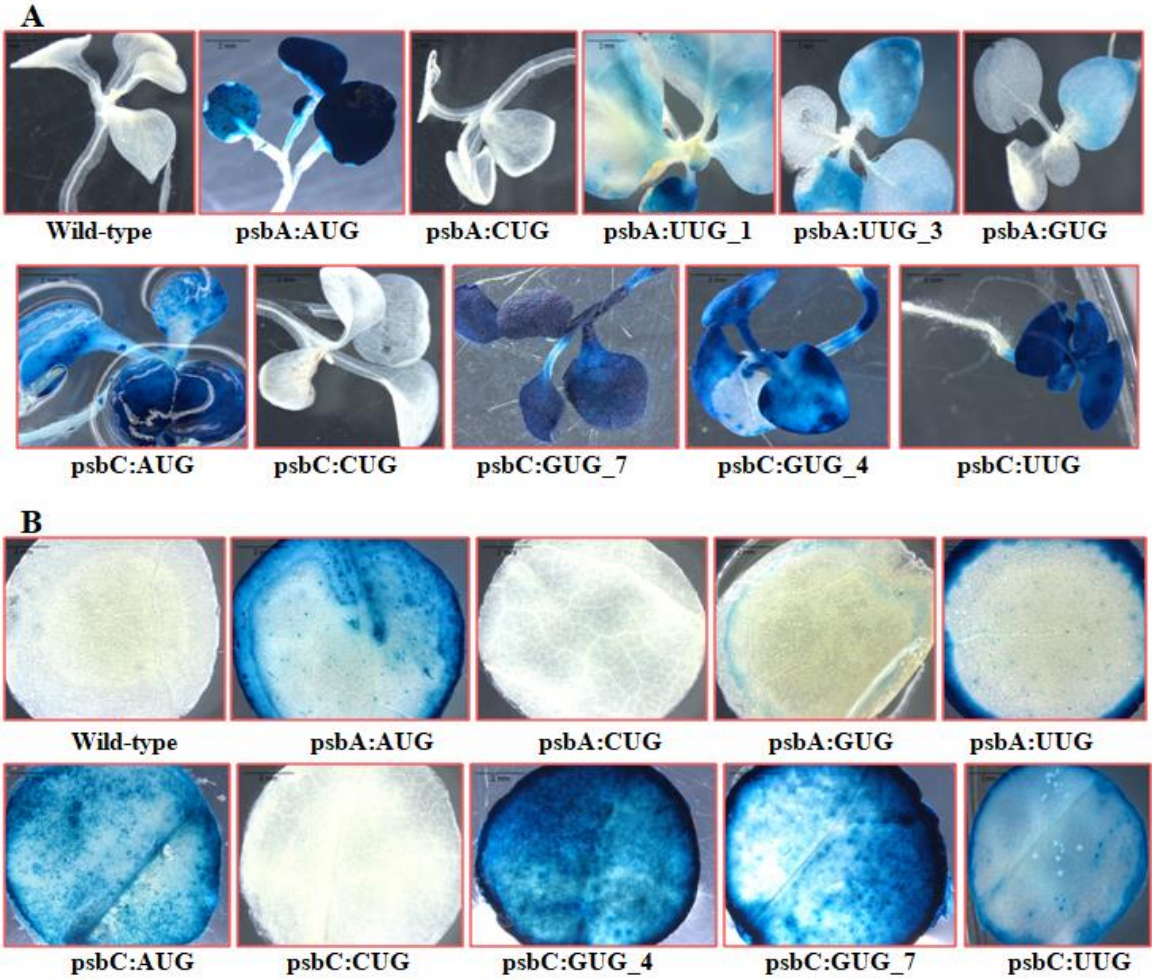

Figure S5. Histochemical staining of β-glucuronidase (GUS) expression in 10 days old seedlings **(A)** and leaf tissues from 5-week-old **(B)** transplastomic homoplasmic lines expressing *uidA* gene with four different start codons which are driven by either psbA or psbC promoters. Numbers 1, 3, 4 and 7 in psbA:UUG_1, psbA:UUG_3, psbC:GUG_4 and psbC:GUG_7, respectively, correspond to independent transplastomic lines of psbA:UUG and psbC:GUG constructs.

C

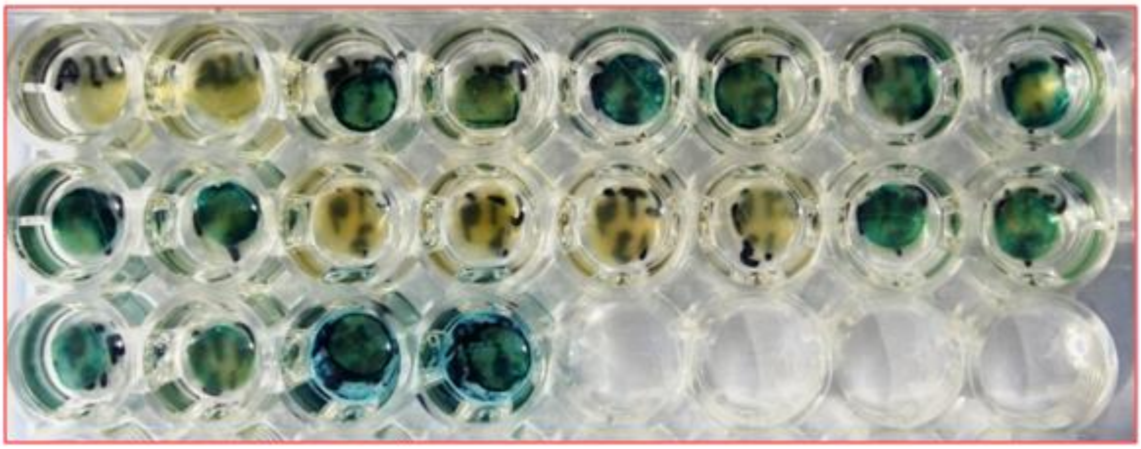

Row 1 represents Nt. Wt (untransformed wild type *Nicotiana tabaccum*), psbA:UUG_1, psbA:UUG_3, and psbA:UUG_4 transplastomic line leaf disc in well no. 1-2, 3-4, 5-6, and 7-8, respectively. **Row 2 represent**s psbA:UUG_7, psbA:CUG_3, psbA:CUG_13, and psbA:GUG_7 in well no. 1-2, 3-4, 5-6, and 7-8, respectively.
Row 3 represents psbA:GUG_12 and psbA:AUG in well no. 1-2, and 3-4, respectively.

D

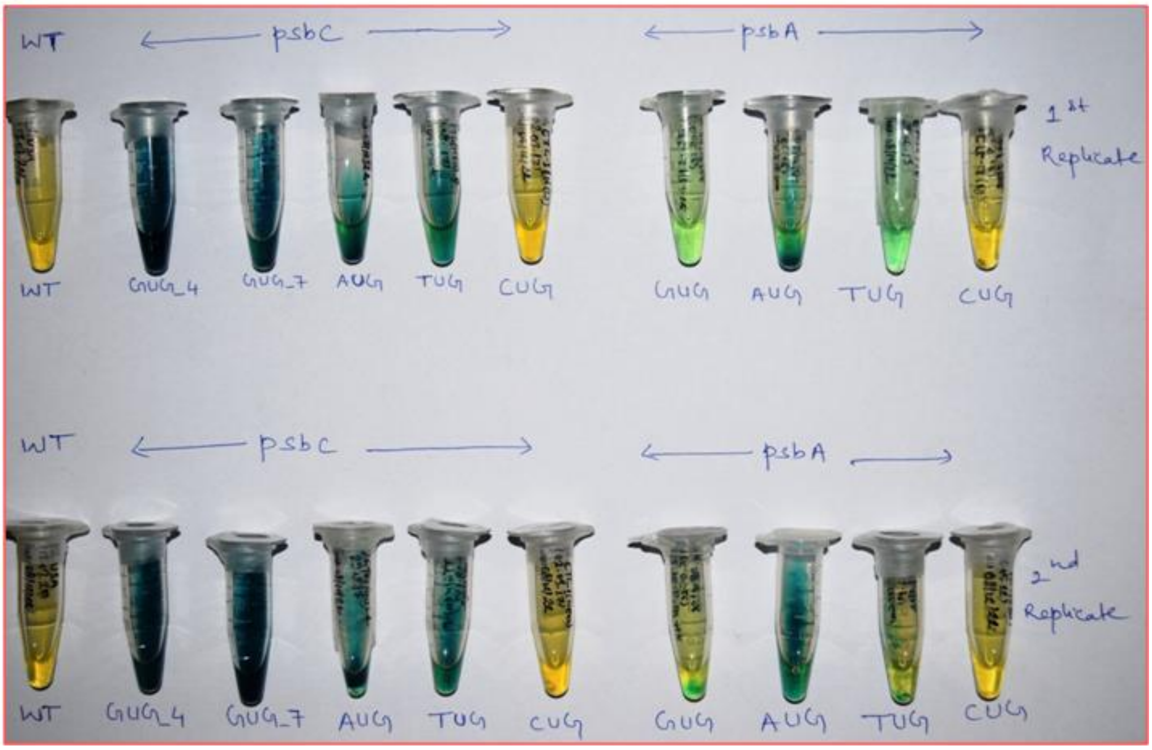

Figure S5. Histochemical staining for β-glucuronidase (GUS) expression. **(C)** Total four independently regenerated lines of psbA:UUG, two lines of psbA:CUG, two lines of psbA:GUG, and one line of psbA:AUG were used for GUS expression initially. Randomly single transplastomic homoplastic lines per construct were chosen for further studies. **(D)** X-Gluc solution turned blue (except in wild type/WT, psbC:CUG, and psbA:CUG expressing plants) when the leaf discs from 5-week-old plants were incubated at 37º C for 24 hours in the substrate.

```
psbAP        4 TTCACAGTTTCCATTCTGAAATGTTCTCTGTACTATAATAAATAGTAAGTGAATCAACTT
psbCP      101 TCCACTGGGTC-AGTCTGGTTGGTTCTTTGCACCTAGTTTTGGTGTAGCAGCTATATTTC
               * *** *  ** * ****    ***** ** **     *     ***   *    *  *

psbAP       64 ACTACTAAAAAAATTAGTA--GACTTCCTCTTCGGAATAGAAATAGCCTATTTCTACATA
psbCP      160 GATTCATCCTCTTTTTTCAAGGGTTTCATAATTGGA--CGTTGAACCCATTTCATATGAT
                 * *        **   *  *  *** *  * ***   *    * **  **  **

psbAP      122 GGGAAAGTCGTGTGCAATGAAAAATGC------AAGCACGATTTGGGGAGA------GGT
psbCP      218 GGGAGTTGCCGGTGTATTGGGCGCTGCTTTGCTATGCGCCATTCATGGTGCTACCGTAGA
               ****    *  *** * **     ***      * ** * ***   ** *        *

psbAP      170 TTTTTCTCTATTGTAACAAGGAATAATTATCTACTCCATCCGA-CTAGTT-CCGGGTTCG
psbCP      278 AAATACTTTATTTGAAGACGGTGATGGTGCAAATACATTCCGTGCTTTTAACCCAACTCA
                  * ** ****  ** * **      *    *  *  ****  **  *  **    **

psbAP      228 AGTCCCGGGCAACCCAT---ATGGAAACTAGAAAGGAGCAATCTGAGTTTTGATTTTTCA
psbCP      338 AG-CCGAAGAAACTTATTCAATGGTCAC-CGCTAACCGCTTTTGGTCCCAAATCTTTGGG
               ** **   * ***  **   ****  **  *  *   **  *  *         ***

psbAP      285 CTCACTTCATTTACAAAATTTTTTGGTTTGGTAAATTT-TGTTGTATGGATATACAACTG
psbCP      396 GTTGCTT--TTTCCAATAAACGTTGGTTACATTTCTTTATGTTATTTGTACCAGTAAC--
                *  ***  *** *** *    ******   *   *** **** * ** *     ***

psbAP      344 TCGGGGCTGGCTTGGTTGACATTGGTATATAGTCTATATTATACTGTTAAATAACAAGCC
psbCP      452 -CGGTTTATGGATGAGTGCTCTTGGAGTA--GTCGGTCTAGCCCTG--AACCTACGTGCC
                ***     *  **  **   ****  **  ***  * *    ***  **   **  ***

psbAP      404 TTCTATTATCTTTCT-AGTTAATACGTGTGCTTGGGAGTCCT-----TGCAATTGAATAA
psbCP      507 TATGACTTCGTTTCTCAGGAAATTCGCGCAGCGGAAGATCCTGAATTTGAGACTTTCTAC
               *   * *   ***** **  *** ** *     *    ****     **  * *   **

psbAP      458 ACCAAGATCTTACCATG
psbCP      567 ACCAAAAATATTCTCTTAAACGAAGGTATTCGCGCTTGGATGGCGGCTCAAGATCAGCCT
               ********  * *  *

psbAP
psbCP      627 CATGAAAACCTTATATTCCCTGAGGAGGTTCTACCACGTGGAAACGCTCTTTAATGCA
```

Supplementary Fig S6. Homology between the tobacco psbC promoter (psbCP) and rice psbA promoter (psbAP) sequences used in the present study to express the *uidA* gene. The -35 and -10 elements are highlighted in blue. Also shown transcript initiation site (red) and start codon (yellow). Note that six N-terminal codons (green) of the *psbC* gene were fused in frame with *uidA* (GUS) to conserve the translation start site and translation initiation. The similarity between psbAP and psbCP sequences is carried out using ExPaSy SIM software with the following parameters. Comparison matrix: BLOSUM62; Number of alignments computed: 20; Gap open penalty: 12; Gap extension penalty: 4. The results showed 45.0% identity.

A

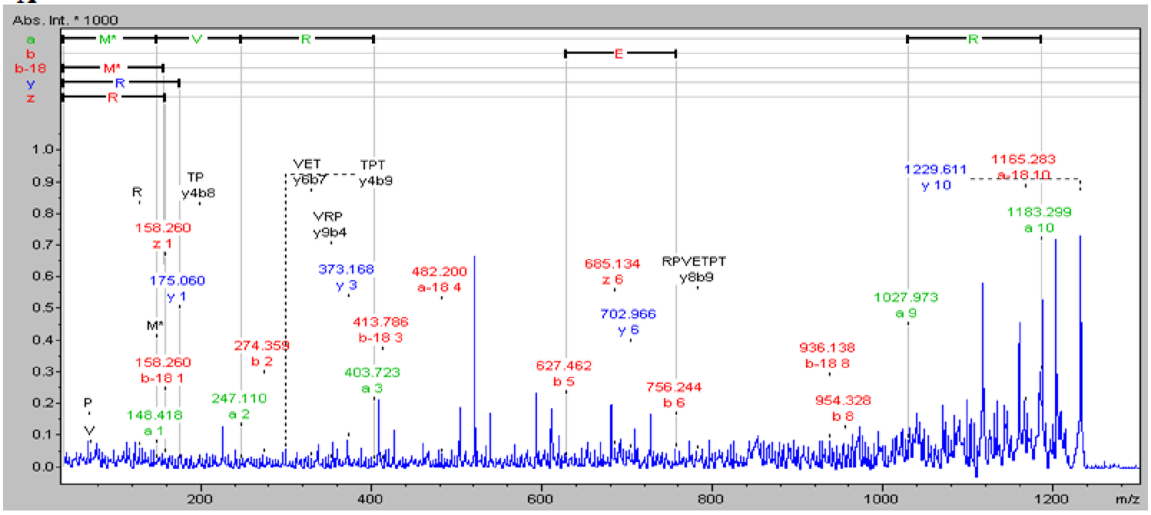

1229.6140 -**M**VRPVETPTR.E + Oxidation (M) + Formylation (M)

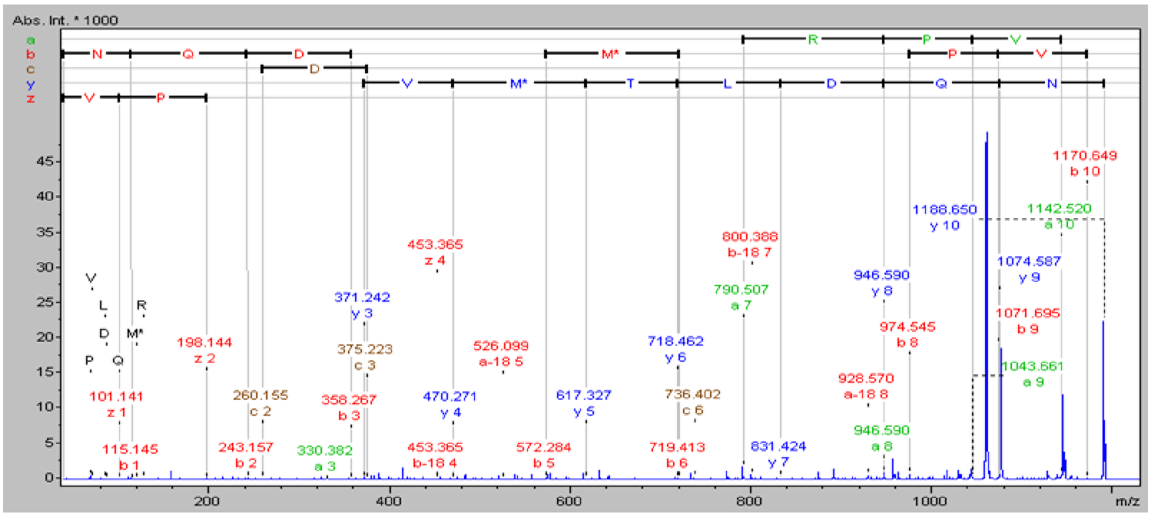

1188.6512 **NQDLTMVRPV.E + Oxidation (M)**

B

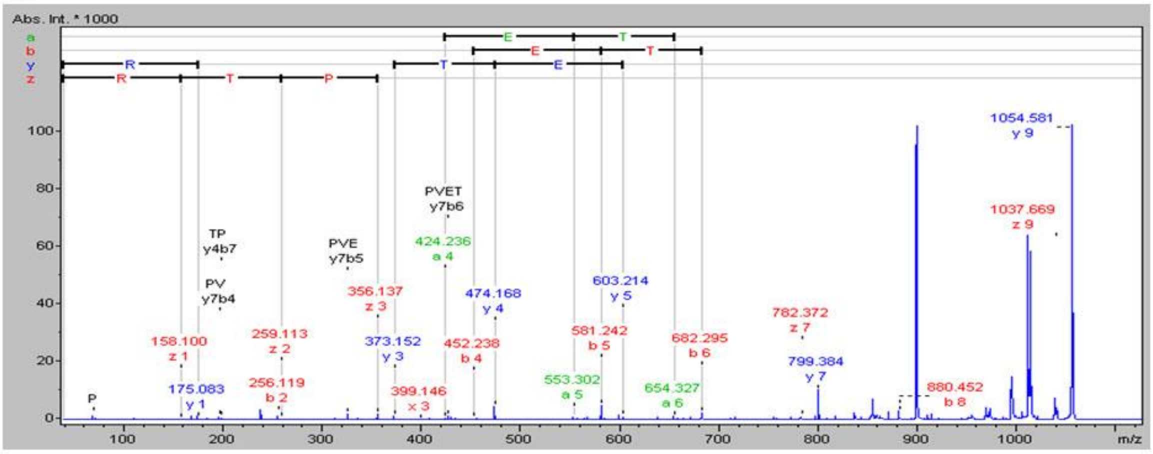

1054.5779 **VRPVETPTR.E**

Supplementary Fig S7. Representative samples of the N-terminal of GUS protein, expressed in transplastomic tobacco plants under psbA promoter having either AUG **(A)** or UUG **(B)** as a start codon, determined by Mass Spectrometry.

C

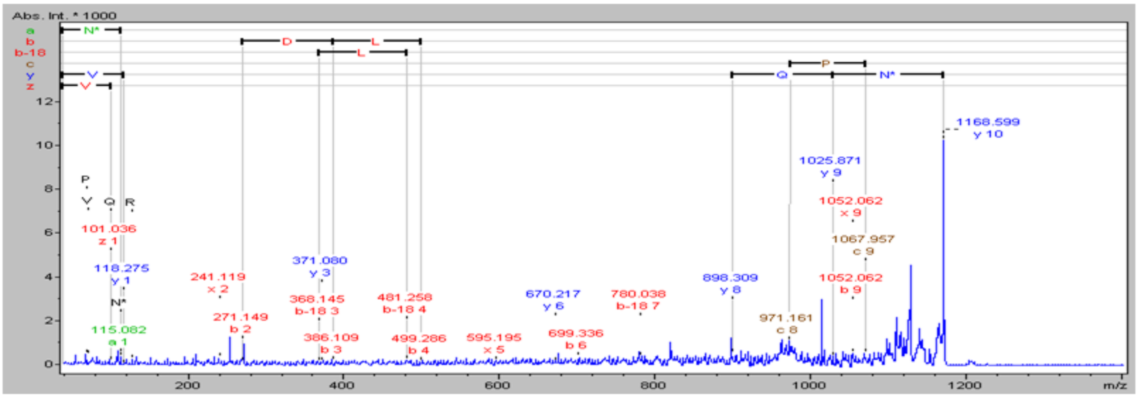

1168.6040 **NQDLTVVRPV.E + Dimethyl (N)**

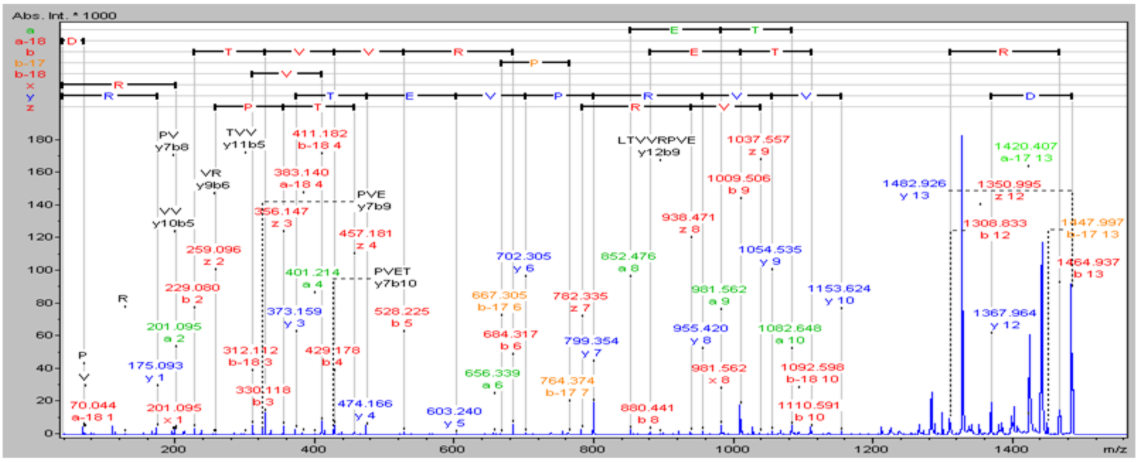

1482.9229 **DLTVVRPVETPTR.E**

D

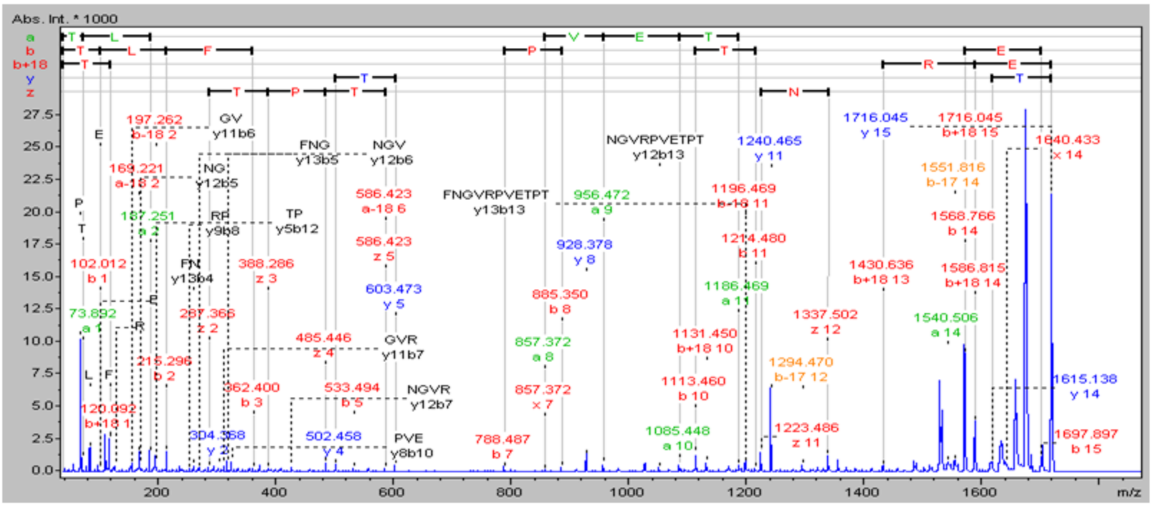

1716.0453 **E.TLFNGVRPVETPTRE.I**

Supplementary Fig S7. Representative samples of the N-terminal of GUS protein, expressed in transplastomic tobacco plants under psbA **(C)** and psbC **(D)** promoters having GUG as a start codon, determined by Mass Spectrometry. Raw data related to protein N-terminal identification is given in Supplementary Information 2 and 3.

Supplementary Table S1. N-terminal of GUS protein expressed in different transplastomic plants determined by mass spectrometry.

**Sample details**	**m/z $(M+H)^+$**	**Sequence coverage (unique peptides)**	**Peptide sequence (MS/MS)***	**Modification (N-terminus)**
Nt. psbA:AUG	1054.6285	28 (16)	VRPVETPTR.E	N-terminal methionine excision
	1188.6512		NQDLT**M**VRPV.E + oxidation (M)	Novel TIS (translation initiation site)
	1229.6140		-**M**VRPVETPTR.E + oxidation (M)	Formylation of N-terminal methionine
Nt. psbA:UUG	1054.5779	13 (7)	VRPVETPTR.E	N-terminal amino acid (leucine) excision
	1496.843		DLT**L**VRPVETPTR	TIS
Nt. psbA:GUG	1168.6040	5 (3)	NQDLT**V**VRPV.E + dimethyl (N)	Novel TIS
	1482.9229		DLT**V**VRPVETPTR.E	TIS
Nt. psbC:GUG	1716.0453	11 (7)	ETLFN**G**VRPVETPT RE.I	TIS / N-terminal amino acid excision

*Asp N and Glu C enzymes were used. Modifications due to carbamidomethyl (C) and oxidation (M) were considered in the analysis. Annotated mass spectrum (MS/MS data) is given in Supplementary Fig. S7.

Supplementary Table S2. Oligonucleotides used to construct transformation vectors with psbC promoter and different start codons.

Primer	Sequence
**psbC-SalI**	**5'CGTAGTGTCGACTACGTCAATTCGAGCTTGCTCG**
**psbCGUS-ATG**	**5'GGGGTTTCTACAGGACGGACTCCATTAAAGAGCGTTTCCATGTGGTAGAACCTCCTC**
**psbCGUS-CTG**	**5'GGGGTTTCTACAGGACGGACTCCATTAAAGAGCGTTTCCAGGTGGTAGAACCTCCTC**
**psbCGUS-GTG**	**5'GGGGTTTCTACAGGACGGACTCCATTAAAGAGCGTTTCCACGTGGTAGAACCTCCTC**
**psbCGUS-TTG**	**5'GGGGTTTCTACAGGACGGACTCCATTAAAGAGCGTTTCCAAGTGGTAGAACCTCCTC**
**GUS3-SacI**	**5'TCAGGTGCTCTGATTGTTTGCCTCCCTGC**
**psbAGUS-CTG/F**	**5'GAATAAACCAAGATCTTACCCTGGTCCGTCCTGTAGAAAC**
**psbAGUS-CTG/R**	**3'CTTATTTGGTTCTAGAATGGGACCAGGCAGGACATCTTTG**
**psbAGUS-GTG/F**	**5'GAATAAACCAAGATCTTACCGTGGTCCGTCCTGTAGAAAC**
**psbAGUS-GTG/R**	**3'CTTATTTGGTTCTAGAATGGCACCAGGCAGGACATCTTTG**
**psbAGUS-TTG/F**	**5'GAATAAACCAAGATCTTACCTTGGTCCGTCCTGTAGAAAC**
**psbAGUS-TTG/R**	**3'CTTATTTGGTTCTAGAATGGAACCAGGCAGGACATCTTTG**
